# Mucosal signatures of pathogenic T cells in HLA-B*27^+^ anterior uveitis and axial spondyloarthritis

**DOI:** 10.1172/jci.insight.174776

**Published:** 2024-07-18

**Authors:** Michael A. Paley, Xinbo Yang, Lynn M. Hassman, Frank Penkava, Lee I. Garner, Grace L. Paley, Nicole Linskey, Ryan Agnew, Paulo Henrique Arantes de Faria, Annie Feng, Sophia Y. Li, Davide Simone, Elisha D.O. Roberson, Philip A. Ruzycki, Ekaterina Esaulova, Jennifer Laurent, Lacey Feigl-Lenzen, Luke E. Springer, Chang Liu, Geraldine M. Gillespie, Paul Bowness, K. Christopher Garcia, Wayne M. Yokoyama

**Affiliations:** 1 Department of Medicine, Washington University School of Medicine, St. Louis, Missouri, USA.; 2 Departments of Molecular and Cellular Physiology and Structural Biology, Stanford University School of Medicine, Stanford, California, USA.; 3 Department of Ophthalmology and Visual Sciences, Washington University School of Medicine, St. Louis, Missouri, USA.; 4 Nuffield Department of Orthopaedics Rheumatology and Musculoskeletal Science, Botnar Research Center,; 5 NDM Research Building, Nuffield Department of Medicine, and; 6 Centre for Immuno-oncology, Nuffield Department of Medicine, University of Oxford, Oxford, United Kingdom.; 7 Department of Genetics and; 8 Department of Pathology and Immunology, Washington University School of Medicine, St. Louis, Missouri, USA.; 9 Howard Hughes Medical Institute, Stanford University School of Medicine, Stanford, California, USA.; 10 Bursky Center for Human Immunology and Immunotherapy Programs, Washington University School of Medicine, St. Louis, Missouri, USA.

**Keywords:** Autoimmunity, Rheumatology, T cell receptor, T cells

## Abstract

HLA-B*27 was one of the first HLA alleles associated with an autoimmune disease, i.e., axial spondyloarthritis (axSpA) and acute anterior uveitis (B27AAU), which cause joint and eye inflammation, respectively. Gastrointestinal inflammation has been suggested as a trigger of axSpA. We recently identified a bacterial peptide (YeiH) that can be presented by HLA-B*27 to expanded public T cell receptors in the joint in axSpA and the eye in B27AAU. While YeiH is present in enteric microbiota and pathogens, additional evidence that pathogenic T cells in HLA-B*27–associated autoimmunity may have had a prior antigenic encounter within the gastrointestinal tract remains lacking. Here, we analyzed ocular, synovial, and blood T cells in B27AAU and axSpA, showing that YeiH-specific CD8^+^ T cells express a mucosal gene set and surface proteins consistent with intestinal differentiation, including CD161, integrin α4β7, and CCR6. In addition, we found an expansion of YeiH-specific CD8^+^ T cells in axSpA and B27AAU blood compared with that from individuals acting as healthy controls, whereas influenza-specific CD8^+^ T cells were equivalent across groups. Finally, we demonstrated the dispensability of TRBV9 for antigen recognition. Collectively, our data suggest that, in HLA-B27–associated autoimmunity, early antigen exposure and differentiation of pathogenic CD8^+^ T cells may occur in enteric organs.

## Abstract 2

Pathogenic CD8 T cells with specificity to YeiH appear to originate from the gut and may distinguish healthy individuals from patients with HLA-B*27-associated inflammation.

## Graphical abstract

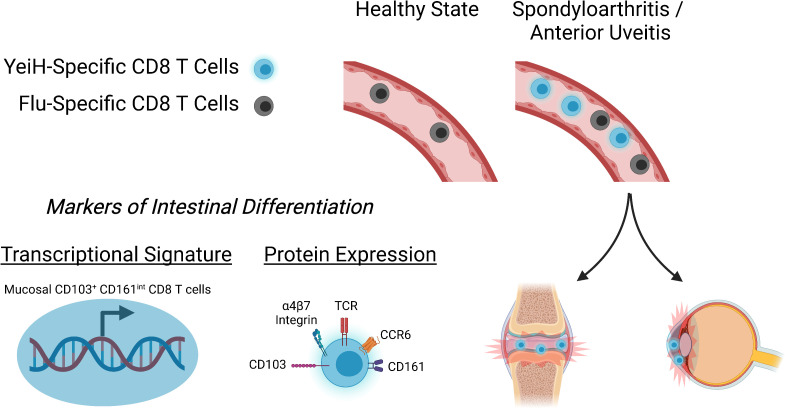

## Introduction

Axial spondyloarthritis (axSpA) is a chronic inflammatory disorder that damages the spine, with occasional involvement of peripheral joints and tendons (1). The greatest genetic risk factor for both radiographic axSpA (r-axSpA) and nonradiographic axSpA (nr-axSpA) is the HLA-B*27 allele; more than 80% of patients are HLA-B*27^+^ compared with less than 10% of the general population (2–4). Many patients with axSpA also have acute anterior uveitis (AAU), and HLA-B*27 increases the risk for developing isolated AAU; 50% of individuals with AAU are HLA-B27^+^ (5, 6). HLA-B*27–associated AAU (B27AAU) presents as sudden onset, unilateral ocular inflammation, with resolution after several weeks (7, 8). Patients with B27AAU are also at high risk (55%–90%) of developing axSpA (6, 9). The shared genetic risk allele of HLA-B*27 and clinical overlap of axSpA and B27AAU suggest a common pathophysiologic mechanism.

HLA-B*27 has been suggested to increase the risk of developing axSpA and B27AAU through various mechanisms. One hypothesis is that HLA-B*27 indirectly causes loss of immune tolerance via its deleterious effect on immune homeostasis and promotion of intestinal inflammation (10–12). Investigators have identified asymptomatic, microscopic inflammation in intestinal biopsies of patients with either r-axSpA or reactive arthritis, the latter being clinically associated with specific intestinal pathogens, including *Shigella* and *Salmonella* (13–15). However, which immune cells or microbial products are responsible for transferring enteric inflammation to the joints in axSpA or eye in B27AAU remain unclear (16). Another hypothesis is that HLA-B*27 presents antigens to pathogenic CD8^+^ T cells in the form of molecular mimicry, whereby CD8^+^ T cells initially respond to foreign antigens, such as from bacteria, but subsequently are stimulated by self-antigens due to cross-reactivity of their T cell receptors (TCRs) (16). This “arthrogenic peptide” hypothesis is supported by our recent identification of axSpA-associated public TCRs that are enriched in the joint in axSpA and eye of B27AAU and can recognize both bacterial and self-peptides presented by HLA-B*27 (17). Moreover, elimination of these public TCRs via TRBV9-targeted depletion leads to disease improvement, at least in some cases (18, 19). One bacterial peptide recognized by these public TCRs is derived from *yeiH*, a gene present in multiple enteric microbes. Whether these expanded CD8^+^ T cells in axSpA and B27AAU have a signature of mucosal immunity remains unknown.

CD161 (*KLRB1*) is expressed on 2 subsets of T cells that are associated with enteric immunity. CD161^hi^CD8^+^ T cells are mucosal-associated invariant T (MAIT) cells that are enriched in enteric organs, such as the liver and intestine; participate in immunity to microbiota; and may play a role in axSpA and B27AAU (20–22). These unconventional T cells recognize 5-OP-RU from bacterial riboflavin biosynthesis in the context of the MHC class I–like molecule MR1 (23). In contrast to MAIT cells, CD8^+^ T cells expressing intermediate levels of CD161 (CD161^int^) are conventional polyclonal αβ T cells that recognize foreign peptides presented by canonical MHC molecules (24). Despite this distinction, CD161^int^CD8^+^ T cells share several properties with MAIT cells. For instance, CD161^int^CD8^+^ T cells are enriched in enteric organs, particularly the colon (24), and contain a distinct transcriptional program that is shared with MAIT cells (25). In addition, CD161 is expressed by a population of CD8^+^ T cells that establish tissue residency within the small intestine (26). However, whether antigen-specific CD161^int^CD8^+^ T cells play a role in axSpA and B27AAU remains unknown.

Here, we report that B27AAU is characterized by an influx YeiH.232-240–specific CD161^int^CD8^+^ T cells into the eye. Moreover, these YeiH.232-240–specific CD8^+^ T cells expressed an intestinal CD8^+^ T cell transcriptional program, were more abundant in axSpA and B27AAU compared with individuals acting as healthy controls, and expressed surface molecules associated with mucosal trafficking and immunity. Collectively, our data suggest that, in HLA-B27–associated inflammation, the early activation of pathogenic T cells may occur in enteric organs.

## Results

We have reported that CD8^+^ T cell clonotypes using TRBV9 were enriched in the joints of individuals with r-axSpA and the eyes of individuals with B27AAU and recognize both bacterial (YeiH.232-240) and self-antigens (17). For that report, we focused on TCRs with the TRBV9 gene segment owing to a historical association of this motif with axSpA (27–29). Recognition of YeiH.232-240 and the self-antigens, however, is not mediated by the CDR1β or CDR2β loops encoded by TRBV9 but rather via 3 amino acids (AIY) genetically encoded in the CDR1α loop of TRAV21 and 4 amino acids in the CDR3β loop [(L/T) (Y/F)ST] that are generated during VDJ recombination. We therefore sought to determine if there could be additional expanded TCRs in B27AAU samples that also recognized YeiH.232-240 and self-antigens.

**Figure 1:**
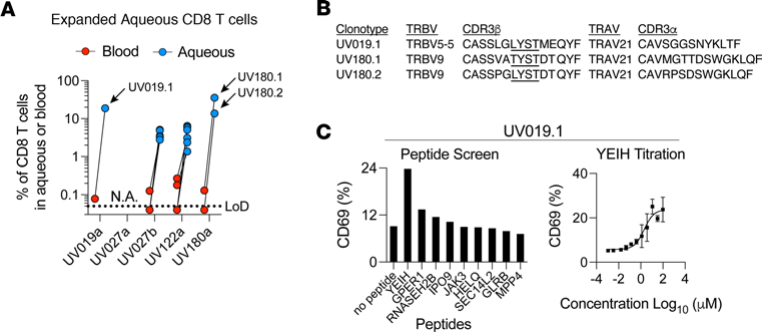
Identification of an expanded TRBV5-5 CD8^+^ T cell clonotype in B27AAU that shares YeiH reactivity. (**A**) Percentage of CD8^+^ T cells in the eye or blood for expanded TCR clones (defined as 10 or more barcodes in the eye). Clones that contain the (L/) ((Y/F)ST CDR3β (UV180.1 and UV180.2) or a similar (UV019.1) CD3β motif paired with AV21 are indicated. The a and b suffixes designate the first and second samplings, respectively. The level of detection (LoD) of blood samples is indicated by a dashed line and is the median proportion of a singleton clonotype from all blood samples. (**B**) TRAV and TRBV gene segment usage and CDR3α and CDR3β sequences for expanded ocular CD8^+^ T cell clonotypes with the (L) () (Y/F)ST CDR3β motif paired with TRAV21. (**C**) Peptide stimulation of the UV019.1 clonotype. SKW-3 T cells transfected with the UV019.1 TCR were cocultured with K562 cells transfected with HLA-B*27:05 in the presence or absence of 100 μM peptides from indicated genes. Samples were run in triplicate.

We first augmented our initial single-cell TCR-sequencing data set (17) with whole genome expression data. After combining our TCR and RNA expression data sets, we divided T cell clonotypes into CD4^+^ and CD8^+^ T cells based on cluster expression of *CD4* and *CD8A* (see Methods) and reexamined the expanded CD8^+^ T cell clonotypes in the eye. Four of the 5 B27AAU ocular samples contained at least 1 CD8^+^ T cell clonotype expanded with 10 or more cells (Figure 1A and Supplemental Table 1; supplemental material available online with this article; https://doi.org/10.1172/jci.insight.174776DS1). The one exception was the ocular sample that was not collected at the initial onset of a flare, but rather after a month-long course of oral corticosteroids (UV027a). Consistent with our prior observations (17), when compared with the frequency in the blood, the expanded ocular CD8^+^ T cell clonotypes were 10- to 100-fold enriched in the eye.

We previously reported that clonotypes UV180.1 and UV180.2 were TRBV9^+^ TCRs with the (L/T) (Y/F)ST CDR3β motif paired with TRAV21 (17) (Figure 1B). Within our focused collection of expanded ocular CD8^+^ T cells, we found that, although clonotype UV019.1 utilized TRBV5-5 instead of TRBV9, the CDR3β contained an LYST motif and was paired with TRAV21, suggesting similar antigen specificity (Figure 1B). We therefore tested whether UV019.1 would respond to any of the described cognate bacterial or self-antigens. SKW-3 T cells expressing the UV019.1 TCR were exposed to HLA-B*27:05–expressing K562 cells pulsed with various bacterial and self-peptides (17). We found that, similar to TCRs using TRBV9, UV019.1 also reacted to YeiH.232-240 (Figure 1C). Thus, the (L/T) (Y/F)ST CDR3β motif paired with TRAV21 (hereafter referred to as the HLA-B*27 axSpA motif) can recognize the YeiH.232-240 epitope presented by HLA-B*27 independent of TRBV9.

YeiH is expressed by enteric microbiota, such as *E*. *coli*, as well as disease-associated bacteria, such as *Shigella* or *Salmonella*. This suggests that CD8^+^ T cells with the HLA-B*27 axSpA motif may have an initial antigenic stimulus at a mucosal barrier. We therefore sought to define the transcriptional signature of these CD8^+^ T cell clonotypes in axSpA and B27AAU.

**Figure 2:**
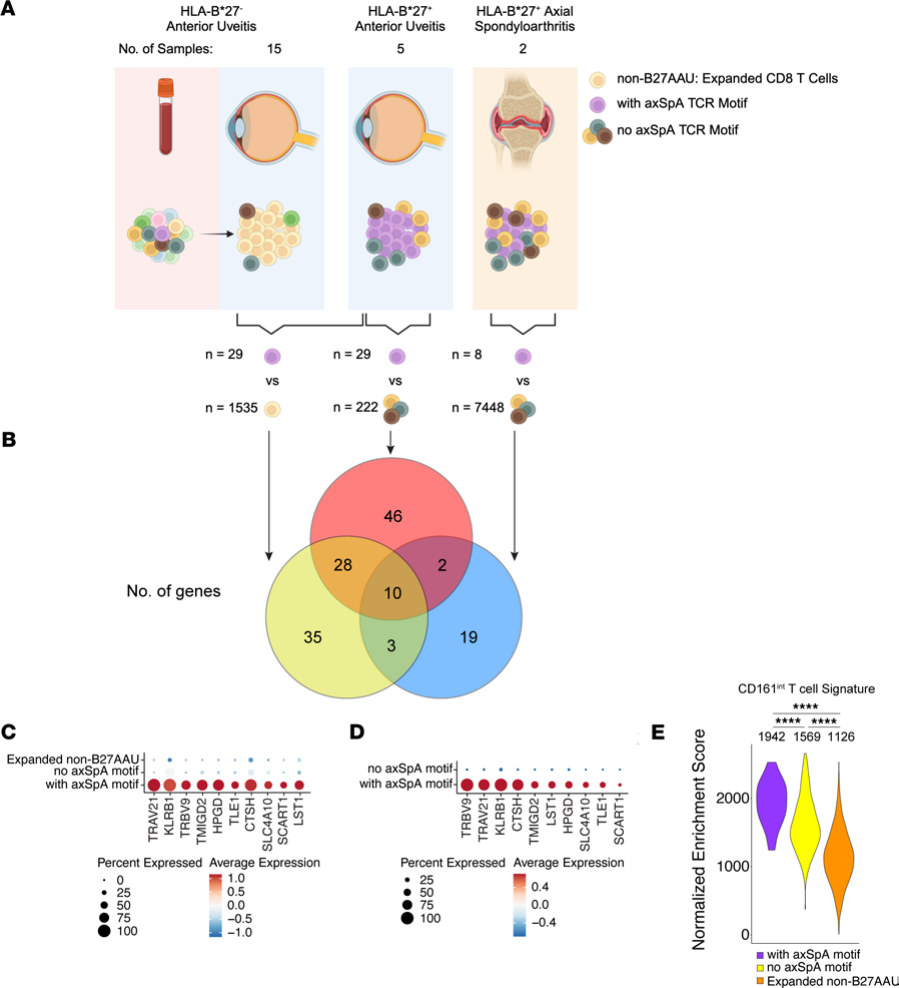
*KLRB1* is expressed by CD8^+^ T cells with the HLA-B*27 axSpA motif. (**A**) Cartoon of 3 transcriptional comparisons to identify differentially expressed genes (DEGs) in CD8^+^ T cells with the HLA-B*27 axSpA motif [defined as pairing of TRAV21 with a () (T) (Y/F)ST CDR3β motif]: (left) clonally expanded ocular CD8^+^ T cells in non-B27AAU vs. CD8^+^ T cells with the HLA-B*27 axSpA motif in B27AAU, (center) ocular CD8^+^ T cells with vs. without the HLA-B*27 axSpA motif, and (right) synovial fluid CD8^+^ T cells with vs. without the HLA-B*27 axSpA motif. (**B**) Number and overlap of DEGs for each transcriptional comparison in **A**. (**C**) Dot plot of the 10 shared DEGs for all 3 transcriptional comparisons for clonally expanded ocular CD8^+^ T cells in non-B27AAU, ocular CD8^+^ T cells without the HLA-B*27 axSpA motif in B27AAU and ocular CD8^+^ T cells with the HLA-B*27 axSpA motif in B27AAU. (**D**) Dot plot of the 10 shared DEGs for all 3 transcriptional comparisons for synovial fluid CD8^+^ T cells with and without the HLA-B*27 axSpA motif. (**E**) Enrichment of the CD161^int^CD8^+^ T cells gene signature for ocular CD8^+^ T cells with (purple) and without (yellow) the HLA-B*27 axSpA motif in B27AAU and clonally expanded ocular CD8^+^ T cells in non-B27AAU (orange). Numbers indicate median enrichment score. ****P < 0.0001, ANOVA. ns, not significant.

To accomplish this, we evaluated single-cell RNA-Seq (scRNA-Seq) data from 14 eye blood–paired samples from participants with HLA-B*27^–^ anterior uveitis and two synovial fluid samples from participants with HLA-B*27^+^ axSpA. We identified differentially expressed genes (DEGs) for CD8^+^ T cells with the HLA-B*27 axSpA motif via 3 comparisons (Supplemental Table 2). First, we compared the gene expression of 29 ocular CD8^+^ T cells with the HLA-B*27 axSpA motif in B27AAU to 1,535 expanded ocular CD8^+^ T cells from other forms of uveitis (non-B27AAU) (Figure 2A and Supplemental Figure 1), identifying 76 upregulated DEGs in ocular CD8^+^ T cells with the HLA-B*27 axSpA motif (Figure 2B). Second, we compared the gene expression of 29 ocular CD8^+^ T cells with the HLA-B*27 axSpA motif to 222 ocular CD8^+^ T cells without the HLA-B*27 axSpA motif from the eyes of participants with B27AAU (Figure 2A), identifying 96 upregulated DEGs in ocular CD8^+^ T cells with the HLA-B*27 axSpA motif (Figure 2B). Third, we compared the gene expression of 8 synovial CD8^+^ T cells with the HLA-B*27 axSpA motif to synovial CD8^+^ T cells without the HLA-B*27 axSpA motif from the joints of participants with axSpA (Figure 2A), identifying 34 upregulated DEGs in synovial CD8^+^ T cells with the HLA-B*27 axSpA motif (Figure 2B). Across these 3 comparisons, 10 genes were consistently upregulated in CD8^+^ T cells with the HLA-B*27 axSpA motif (Figure 2B). As expected, 2 of the 10 DEGs were the TCR gene segments *TRBV9* and *TRAV21*, demonstrating internal consistency in the gene expression analysis. The other 8 DEGs were made up of 3 surface receptors (*KLRB1*, *TMIGD2*, *SCART1*), 1 oxidoreductase involved in prostaglandin metabolism (*HPGD*), 1 transcriptional regulator (*TLE1*), 1 lysosomal protease (*CTSH*), 1 sodium-coupled bicarbonate transporter (*SLC4A10*), and 1 transmembrane adapter protein (*LST1*) (Figure 2, C and D).

The increased expression of *KLRB1* associated with the HLA-B*27 axSpA motif suggested that these cells may reside within the CD161^int^CD8^+^ T cell compartment. To test whether a CD161^int^CD8^+^ T cell gene signature was enriched in CD8^+^ T cells with the HLA-B*27 axSpA motif, we performed single-cell gene set enrichment analysis of a previously reported gene set from CD161^int^CD8^+^ T cells (25). We found that this gene signature was enriched in CD8^+^ T cell clonotypes with the HLA-B*27 axSpA motif compared with other CD8^+^ T cell clonotypes in B27AAU and expanded CD8^+^ T cells in non-B27AAU (Figure 2E). Thus, CD8^+^ T cell clonotypes with the HLA-B*27 axSpA motif express a transcriptional program similar to CD161^int^CD8^+^ T cells.

**Figure 3:**
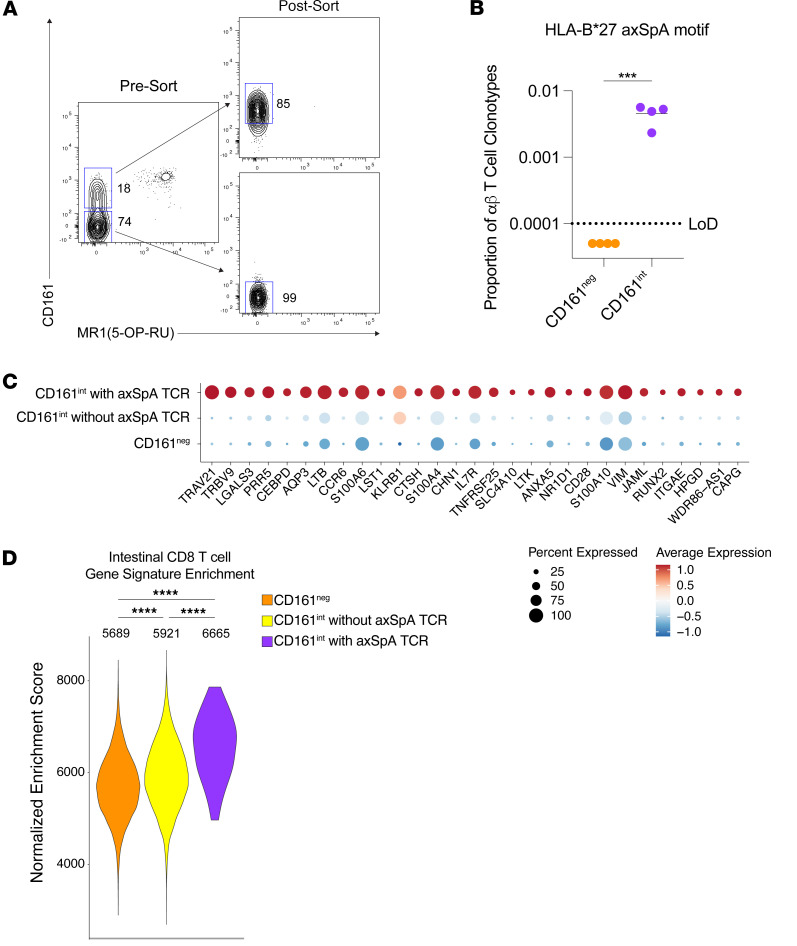
Expanded CD8^+^ T cell clonotypes bearing the HLA-B*27 axSpA motif are found in the CD161^int^CD8^+^ T cell population in the peripheral blood. (**A**) Sort of CD161^int^ and CD161^neg^CD4^–^ T cells for single-cell TCR sequencing. (**B**) Proportion of CD161^neg^ (orange) and CD161^int^ (purple) CD4^–^ αβ T cell clonotypes with the HLA-B*27 axSpA motif. The axSpA motif was uniformly present in the CD161^int^ but not the CD161^neg^ T cell population in 4 samples. ***P < 0.001, ratio paired *t* test. (**C**) Dot plot of DEGs for clonotypes with the HLA-B*27 axSpA motif compared with CD161^int^CD4^–^ αβ T cells without the HLA-B*27 axSpA motif and CD161^neg^CD4^–^ αβ T cells. (**D**) Single-cell gene set enrichment analysis of CD103^+^ intestinal CD8^+^ T cells in CD161^neg^CD4^–^ αβ T cells (orange) and CD161^int^CD4^–^ αβ T cells with (purple) or without (yellow) the HLA-B*27 axSpA motif. Numbers indicate median enrichment score. ****P < 0.0001, ANOVA.

Given the *KLRB1* expression and a CD161^int^ gene signature upregulated in clonotypes with the HLA-B*27 axSpA motif, we next examined whether the expanded ocular CD8^+^ T cell clonotypes were preferentially found in circulating CD161^int^CD8^+^ T cells in the blood. For the 4 participant samples with expanded ocular CD8^+^ T cell clonotypes (UV019a, UV027b, UV122a, and UV180a), we sorted MR1^–^CD4^–^CD3^+^ T cells into CD161^int^ and CD161^neg^ population for scRNA-Seq and TCR sequencing (Figure 3A). This sorting strategy was designed to remove MAIT cells and CD4^+^ Th17 cells, both of which also express CD161 (25). In addition, we did not restrict this analysis to only CD8^+^ T cells, as activated CD8^+^ T cells may downregulate surface CD8 expression (30). We examined whether CD161 expression provided a general enrichment for circulating T cells bearing the HLA-B*27 axSpA motif. We found that, in all 4 B27AAU samples, clonotypes with the HLA-B*27 axSpA motif were found in the CD161^int^ T cell population but were below the limit of detection in the CD161^neg^ population (Figure 3B). These data suggest that pathogenic T cells associated with axSpA and B27AAU are preferentially located within the CD161^int^CD8^+^ T cell population.

Given than the UV019.1 TCR incorporated TRBV5-5 and not TRBV9, suggesting that the CDR1β and CDR2β encoded by TRBV9 are not required for antigen recognition or HLA-B*27 binding, we next evaluated the TCR sequences with the HLA-B*27 axSpA motif in CD161^int^ T cells to determine the frequency of TRBV9 usage. Consistent with prior reports that TRBV9 is associated with the HLA-B*27 axSpA motif, 75% of TCRs with the HLA-B*27 axSpA motif (15 of 20) utilized TRBV9 (Table 1). Several TCRs with the axSpA motif, however, used TRBV5-4, TRBV5-5, or TRBV7-3. These alternative TRBVs were found in 3 of the 4 patients. These data suggest that alternative TRBV gene segments can be used to generate the axSpA TCR motif and may be more prevalent than previously appreciated.

We next compared the transcriptional signature of sorted blood CD161^neg^ and CD161^int^ T cell clonotypes with and without the HLA-B*27 axSpA motif. We found 29 DEGs upregulated in CD161^int^ T cell clonotypes with the HLA-B*27 axSpA motif (Figure 3C). As expected, based on the TCR sequencing, expression of *TRAV21* and *TRBV9*, as measured by total genome expression, was enriched in T cells with the HLA-B*27 axSpA motif. We noted increased expression of *HPGD*, *CTSH*, *SLC4A10*, and *LST1* in T cells with the HLA-B*27 axSpA motif. We also noted upregulation of several surface molecules (*CCR6*, *CD28*, *AQP3*, *ITGAE*, *LGALS3*) and the transcription factor (*CEBPD*) (Figure 3C). This pattern of gene expression has been reported in CD8^+^ T cells at mucosal surfaces (25, 26, 31).

Prior work has suggested 2 subsets of intestinal memory CD8^+^ T cells in humans that are distinguished by the surface expression of CD103 (*ITGAE*) (26). We therefore tested whether the gene signature from either population was enriched in circulating CD8^+^ T cells bearing the axSpA motif, which would suggest transcriptional imprinting from an initial antigen encounter. We found that the transcriptional signature of intestinal CD103^+^CD8^+^ T cells was enriched in CD161^int^ T cells with the axSpA motif compared with other CD161^int^ and CD161^neg^ T cells (Figure 3D). This suggests that pathogenic CD8^+^ T cells in axSpA and B27AAU may retain a transcriptional program from antigen stimulation and differentiation events in the intestine.

As the HLA-B*27 motif was found in CD161^int^ T cells, we investigated whether the CD161^int^ CD8^+^ T cell population was globally dysregulated in participants with HLA-B*27–associated inflammation. We found that participants with B27AAU and/or HLA-B*27^+^ axSpA had equivalent levels of CD161^int^CD8^+^ T cells in the peripheral blood compared with HLA-B*27^+^ individuals acting as healthy controls (Supplemental Figure 2A). We next examined the expression of selected surface molecules from the HLA-B*27 axSpA motif DEGs, i.e., *CCR6*, *CD28*, and *ITGAE* (CD103) (Figure 3C), in total CD161^int^CD8^+^ T cells. We found higher expression of CCR6 in the CD161^int^CD8^+^ T cell population (Supplemental Figure 2B), consistent with prior reports (25). CD161^int^CD8^+^ T cells also had higher expression of CD103 (Supplemental Figure 2C), similar to the CD161 and CD103 coexpression of intestinal memory CD8^+^ T cells (26)**.** We did not find any differences in these populations between healthy individuals and participants with HLA-B*27^+^ axSpA and/or B27AAU. In contrast to CCR6 and CD103, we found a lower protein expression of CD28 in CD161^int^CD8^+^ T cells (Supplemental Figure 2D), despite increased mRNA expression of *CD28* in CD161^int^ T cells with the HLA-B*27 motif. Thus, the transcriptional signature of CD8^+^ T cells with the axSpA motif partially aligns with the protein expression of CD161^int^CD8^+^ T cells.

**Figure 4:**
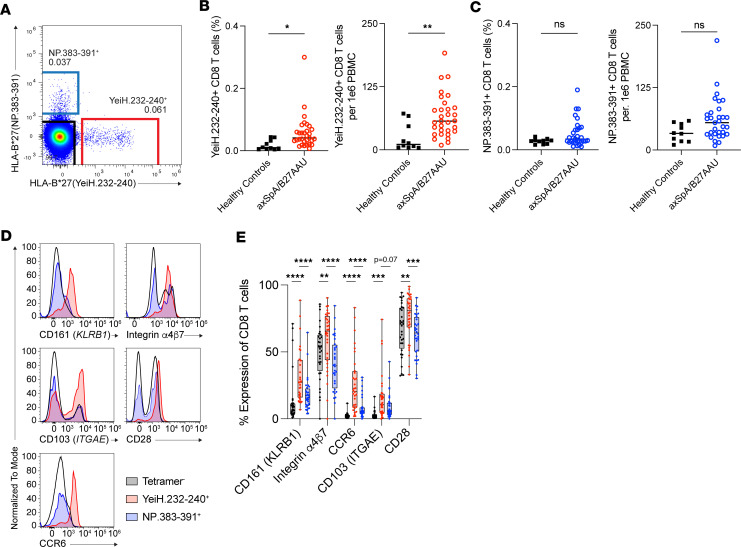
YeiH.232-240–specific CD8^+^ T cells express markers of enteric trafficking and differentiation. (**A**) Identification of HLA-B*27(YeiH.232-240)^+^ and HLA-B*27(NP.383-391)^+^ CD8^+^ T cells by flow cytometry in the peripheral blood of a participant with B27AAU. Cells are gated on CD4^–^CD19^–^Va7.2^–^Va24.Ja18^–^CD3^+^CD8^+^ T cells. (**B**) Quantification of HLA-B*27(YeiH.232-240)^+^ CD8^+^ T cells in peripheral blood from HLA-B*27^+^ individuals acting as healthy controls (*n* = 10) and participants with HLA*B27^+^ axSpA and/or B27AAU (*n* = 31). Tetramer^+^ cells are shown both as a percentage of all CD8^+^ T cells as well as the number of cells per 1 million PBMCs. (**C**) Quantification of HLA-B*27(NP.383-391)^+^ CD8^+^ T cells in the same groups as in **B**. (**D**) Expression of CD161, integrin α4β7, CCR6, CD103, and CD28 on HLA-B*27(YeiH.232-240)^+^ (red), HLA-B*27(NP.383-391)^+^ (blue), and tetramer^–^ (black) CD8^+^ T cells in participants with B27AAU and/or axSpA. (**E**) Quantification of **D** (*n* = 14). *P < 0.05; **P < 0.01; ***P < 0.001; ****P < 0.0001, Mann-Whitney test (**B** and **C**) or 2-way ANOVA with Dunnett’s multiple comparisons test (**E**).

As both the TRBV9 TCRs reported previously (17) and the TRBV5-5 TCR reported here (UV019.1) demonstrated signaling to the YeiH.232-240 peptide, we sought to characterize CD8^+^ T cells with this specificity. We first determined whether we could detect and phenotype YeiH.232-240–specific CD8^+^ T cells in the peripheral circulation by flow cytometry in HLA-B*27^+^ individuals acting as healthy controls and participants with HLA-B*27^+^ axSpA and/or B27AAU. As a comparative population, we examined CD8^+^ T cells specific to the influenza epitope NP.383-391 presented by HLA-B*27 as well as tetramer^–^ CD8^+^ T cells (Figure 4A). Both YeiH.232-240–specific and NP.383-391–specific CD8^+^ T cells had a low frequency in the peripheral blood, e.g., often less than 0.1% of all CD8^+^ T cells or less than 100 cells per million PBMCs (Figure 4, B and C). Despite this rarity, we found an increased frequency of YeiH.232-240–specific CD8^+^ T cells, but not NP.383-391–specific CD8^+^ T cells, in the peripheral blood of participants with HLA-B*27–associated inflammation over HLA-B*27^+^ individuals acting as healthy controls (Figure 4, B and C). Moreover, this increase in YeiH.232-240–specific CD8^+^ T cells was found in participants with HLA-B*27^+^ axSpA alone or B27AAU alone (*P* < 0.05, Kruskal-Wallis multiple-comparison testing), demonstrating that expansion of YeiH.232-240–specific CD8^+^ T cells in the peripheral blood is a shared feature of HLA-B*27–associated inflammation (Supplemental Figure 3A).

We next asked whether the frequency of YeiH.232-240–specific CD8^+^ T cells changed with disease activity. As disease activity scores for axSpA are based on subjective patient self-assessments and are therefore confounded by medical comorbidities that lead to chronic pain and dysfunction (32), we focused on B27AAU flares as a more objective measure of disease activity. Standard treatment of B27AAU flares requires several weeks of topical steroids (33), suggesting a prolonged inflammatory stimulus. We therefore measured YeiH.232-240–specific CD8^+^ T cells in the blood within 35 days of a B27AAU flare (based on symptom onset) or during disease quiescence of B27AAU. We found that the frequency of YeiH.232-240–specific CD8^+^ T cells was numerically greater during disease quiescence compared with a recent or active B27AAU flare; however, this did not reach statistical significance (Supplemental Figure 3B). In contrast, the frequency of NP.383-391–specific CD8^+^ T cells was numerically unchanged between active and quiet B27AAU (Supplemental Figure 3C). This analysis was potentially confounded by medication usage, as there were numerically higher rates of NSAID and TNF inhibitor usage during quiescent disease (Supplemental Table 3).

We next examined YeiH.232-240 tetramer^+^ CD8^+^ T cells for surface expression of DEGs associated with the axSpA motif (Figure 3C) (*KLRB1* [CD161], *CCR6*, *CD28*, and *ITGAE* [CD103]) as well as integrin α4β7 (lymphocyte Peyer’s patch adhesion molecule-1 [LPAM-1]), which is involved in trafficking to mucosal surfaces and is a therapeutic target in inflammatory bowel disease (34, 35). For participants with B27AAU and/or axSpA, YeiH.232-240–specific CD8^+^ T cells expressed higher levels of CD161 compared with NP.383-391–specific and tetramer^–^ CD8^+^ T cells (Figure 4, D and E), consistent with our finding that the HLA-B*27 axSpA motif is preferentially found in the CD161^int^ T cell population (Figure 3B). Moreover, YeiH.232-240–specific CD8^+^ T cells also had higher expression of CCR6 and CD103 (Figure 4, D and E), consistent with differentiation into CD161^int^CD8^+^ T cells (Supplemental Figure 2, B and C). Compared with NP.383-391–specific CD8^+^ T cells, YeiH.232-240–specific CD8^+^ T cells expressed higher levels of CD28 (Figure 4, D and E), consistent with gene expression analysis (Figure 3C). Finally, expression of integrin α4β7 was higher in YeiH.232-240–specific CD8^+^ T cells, consistent with a prior episode of intestinal activation (Figure 4, D and E). In HLA-B*27^+^ individuals acting as healthy controls, YeiH.232-240–specific CD8^+^ T cells also displayed a numerically higher average expression of CD161, integrin α4β7, CCR6, CD103, and CD28 compared with tetramer^-^ CD8^+^ T cells, although this did not reach statistical significance (Supplemental Figure 4). Thus, YeiH.232-240–specific CD8^+^ T cells express surface proteins consistent with mucosal trafficking and differentiation.

**Figure 5:**
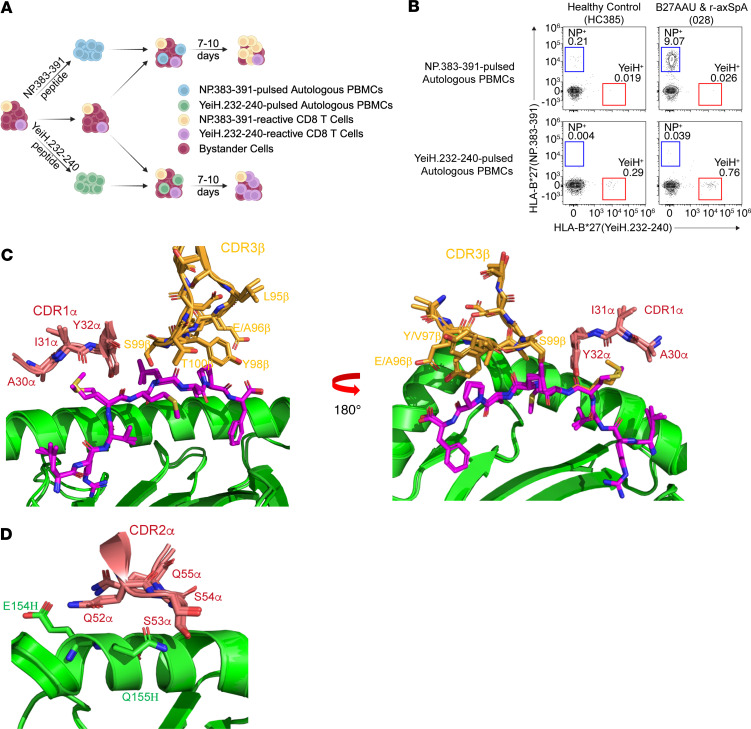
Peptide expansion and tetramer sorting identifies alternative TCR motifs for recognition of YeiH.232-240. (**A**) Cartoon of in vitro expansion of CD8^+^ T cells specific for NP.383-391 or YeiH.232-240. PBMCs are pulsed with NP.383-391 or YeiH.232-240 to generate “stimulators,” which are then combined with autologous “responder” PBMCs and cultured for 7–10 days to allow for expansion of NP.383-391 or YeiH.232-240–specific CD8^+^ T cells. (**B**) Representative flow cytometry of PBMCs from an individual acting as a healthy control (left) or participant with B27AAU and/or axSpA (right) after 8 days of culture with NP.383-391–pulsed (top) or YeiH.232-240–pulsed (bottom) autologous PBMCs. Plots show antigen-specific expansion of tetramer^+^ CD8^+^ T cells. Plots are representative of 4 separate experiments. (**C**) Side view of detailed peptide engagement through superimposed 026.1/135.1 TCR CDR1α and CDR3β. The TCRα chain is colored deep salmon red, the TCRβ chain is colored bright orange, the peptide is colored magenta, and the HLA-B*27:05 is colored green. (**D**) Side view of interactions between 026.1/135.1 TCR CDR2α and HLA-B*27:05. The TCRα chain is colored deep salmon red, and the HLA-B*27:05 is colored green.

We sought to verify that the HLA-B*27(YeiH.232-240) tetramers detect CD8^+^ T cells with the HLA-B*27 axSpA motif. As the frequency of these CD8^+^ T cells is extremely low in the blood, we enrolled a new cohort of HLA-B*27^+^ participants with axSpA and B27AAU and screened them for higher frequencies of YeiH.232-240–specific CD8^+^ T cells via tetramer, i.e., greater than 75 YeiH.232-240–specific CD8^+^ T cells per million PBMCs. In addition, we sought to expand the cells in vitro in order to have a sufficient quantity for single-cell TCR sequencing. To achieve this, we cultured PBMCs from HLA-B*27^+^ individuals acting as healthy controls or participants with B27AAU and/or axSpA with peptide-pulsed autologous PBMCs for 7–10 days (Figure 5A). With this method, we were able to amplify NP.383-391–specific CD8^+^ T cells using NP.383-391–pulsed PBMCs or YeiH.232-240–specific CD8^+^ T cells using YeiH.232-240–pulsed PBMCs, demonstrating antigen-specific expansion (Figure 5B). Of note, there was variability from participant to participant, and YeiH.232-240–specific CD8^+^ T cells remained at low frequency. We then selected PBMCs from 5 HLA-B*27^+^ individuals acting as healthy controls and 5 participants with B27AAU and/or axSpA to stimulate with the YeiH.232-240 peptide, culture for 8 days, and then sort on HLA-B*27(YeiH.232-240) tetramer^+^ CD8^+^ T cells. To minimize contamination from nonspecific binding, we sorted on CD8^+^ T cells that dual stained for both PE-conjugated and APC-conjugated HLA-B*27(YeiH.232-240) tetramers. This strategy was designed to obtain TCRs that had both proliferated in response to peptide stimulation as well as bound the HLA-B*27(YeiH.232-240) tetramer. In total, we sorted approximately 3,000 HLA-B*27(YeiH.232-240) tetramer^+^ CD8^+^ T cells for single-cell TCR sequencing and recovered 17 TCRs from 238 cells (Table 2). These TCRs were from 1 of the 5 healthy participants and 5 of 5 participants with B27AAU and/or axSpA (Table 2).

We made several observations from this set of TCRs. First, 5 (29%) of the TCRs contained the canonical HLA-B*27 axSpA motif, indicating that the HLA-B*27(YeiH.232-240) tetramer can detect pathogenic CD8^+^ T cells. These TCRs were found in 4 of the 5 participants with B27AAU and/or axSpA and utilized TRBV5-4, TRBV5-5, or TRBV9, again demonstrating the dispensability of TRBV9 for YeiH.232-240 antigen recognition. Second, 12 (71%) of the TCRs contained TRAV21, suggesting that the TRAV21 CDR1α may be a common structural solution for YeiH.232-240 peptide recognition. Third, 6 (35%) of the TCRs contained a CDR3β motif of YYST, VYST, SYST, or GYST paired with TRAV21, similar to the LYST motif previously described (17). Finally, 5 (29%) of the TCRs contained no discernible similarity to the HLA-B*27 axSpA motif, suggesting additional structural solutions for YeiH.232-240 recognition. The sole TCR from a healthy participant sample (HC296) was included in this final category (Table 2).

To visualize how TCRs engage YeiH-HLA-B*27 with alternative TRBVs or CDR3β motifs, we modeled 2 TCRs (026.1 and 135.1; Table 2) based on the previously reported TCR-pHLA-B*27 structures (PDB: 8CX4 and 7N2Q) (17). Both TCRs feature the YSTDTQ CDR3β motif, which forms extensive interactions with YeiH.232-240 position 5 to position 9 [P5–P9] residues. The P4 Met of YeiH.232-240 peptide (P1–P4) is mostly engaged by TRAV21 Tyr at P32 within CDR1α (Figure 5C). The CDR2α also formed dense interactions with the HLA-B*27 a2 helix, suggesting the importance of TRAV21 in both peptide recognition and HLA-B*27 binding (Figure 5D). Collectively, these models and our previously reported TCR-peptide-HLA-B*27 structures help to explain the conserved TRAV21 pairing with the pathogenic CDR3β motif for peptide recognition.

Our previous structures of TCR recognition of YeiH.232-240 demonstrated an interaction between L97b or T97b and P6 and P8 of the YeiH.232-240 peptide (17). Prior reports using TCRb sequencing, however, suggested some variability at P97 in the CDR3β motif (36), with multiple amino acids able to substitute for Leu or Thr, e.g., Ile, Val, Arg, Tyr, or Glu. In our modeling of the 026.1 TCR, the Tyr at P97 makes contact with neither the YeiH.232-240 peptide nor HLA-B*27 (Figure 5C), which offers a structural rational for amino acid variability at this position.

In our previous single-cell TCR-sequencing experiment, we noted wide variability of the CDR3α sequences for pathogenic TCRs. In our model, we found that the 026.1 TCR makes a few contacts with HLA-B*27 α1 helix but none with the YeiH.232-240 peptide, consistent with the previously described AS4.2-YEIH-HLA-B*27 and AS8.4-YEIH-HLA-B*27 structures (17), reinforcing the minimal effect of the CDR3α on peptide recognition.

Finally, we examined the structural basis for usage of non-TRBV9 gene segments. The 135.1 TCR utilizes alternative TRBV5-1-YSTDTQ-TRBJ2.3b chain. Similar to the TRBV9^+^ pathogenic TCRs, TRBV5-1 CDR1β and CDR2β were not involved in YeiH peptide or HLA-B*27 interaction. Collectively, these data emphasize how the CDR1α and CDR2α (encoded by TRAV21) and the CDR3β form the structural kernel for peptide-HLA-B*27 recognition and provide a structural rationale for finding non-TRBV9 pathogenic TCRs in participants with B27AAU and/or axSpA.

## Discussion

Here, we report multiple TCRs with TRAV21 paired with a CDR3β sequence LYST (the HLA-B*27 axSpA motif) that can recognize YeiH.232-240 independent of TRBV9. We also demonstrated the ability to detect YeiH.232-240–specific CD8^+^ T cells by flow cytometry and that HLA-B*27(YeiH.232-240) tetramers identify a mixture of TCRs with and without the HLA-B*27 axSpA motif. We identified several core genes expressed by CD8^+^ T cells with the HLA-B*27 axSpA motif, including *KLRB1* (CD161), and subsequently validated that the HLA-B*27 axSpA motif is preferentially found in the CD161^int^ population. We found that YeiH.232-240–specific CD8^+^ T cells are expanded in peripheral blood of individuals with HLA-B*27–associated inflammation compared with individuals acting as healthy controls. Finally, we confirmed that YeiH.232-240–specific CD8^+^ T cells display markers of intestinal T cells, such as CD161, integrin α4β7, CCR6, and CD103. Thus, these data are consistent with a model of T cell activation by enteric bacteria followed by egress from the gut into the blood to ultimately promote inflammation in the joint in axSpA and eye in B27AAU, respectively.

In multiple prior studies, TCRb sequencing has identified the TRBV9 gene segment as part of a TCR motif associated with axSpA (27–29, 37). The amino acids that confer specificity to YeiH and several autoantigens, however, are not genetically encoded by TRBV9 (17), raising the question as to whether TRBV9 is required for antigen recognition. Here, we identified a TRBV5-5 TCR that has undergone clonal expansion and tissue-specific recruitment to the eye in vivo that shares the HLA-B*27 axSpA motif and specificity to YeiH, demonstrating that TRBV9 is not required for the potential to mediate HLA-B*27–associated inflammation. As a result, the preferential use of TRBV9 with the HLA-B*27 axSpA motif remains unexplained. The predominant use of TRBV9 in the HLA-B*27 axSpA motif may arise from recombination bias of TRBV9 with TRBJ2-3 (which encodes the serine and threonine of the (L/T) (Y/F)ST CDR3β motif), favorable thymic selection on HLA-B*27, or potentially unique structural properties of TRBV9 that indirectly facilitate antigen recognition.

The development of a TRBV9-depleting therapy to eliminate pathogenic CD8^+^ T cells in axSpA and induce disease remission in a single patient offered proof of concept for potential novel methods to achieve disease control (18). Our TCR sequencing, however, led us to identify several additional TCRs with the HLA-B*27 axSpA motif with TRBV5-4, TRBV5-5, or TRBV7-3, via unbiased TCR sequencing of aqueous fluid cells, sorting on CD161^int^ T cells, or performing in vitro expansion followed by tetramer-based sorting of YeiH-specific CD8^+^ T cells. These non-TRBV9 TCRs were found in the majority of samples that underwent sorting and TCR sequencing. The presence of these alternative TCRs, which would not be eliminated by an TRBV9-depletion strategy, may limit the efficacy of TRBV9 depletion as monotherapy. Instead, TRAV21 depletion or chimeric antigen receptor T cells targeting YeiH.232-240–specific CD8^+^ T cells may have greater response rates.

We found a minority of healthy individuals contained YeiH.232-240–specific CD8^+^ T cells in their blood. How these individuals generated YeiH.232-240–specific CD8^+^ T cells yet remained free of B27AAU or axSpA remains undefined. As the development of B27AAU and/or axSpA likely requires multiple immunological steps, one might hypothesize that there may be differences in the localization, function, or TCR usage of YeiH.232-240–specific CD8^+^ T cells in healthy individuals compared with patients with B27AAU or axSpA. While the data presented here do not definitively answer this question, our tetramer-based sort and sequencing identified alternative TCR sequences that recognize YeiH.232-240 and, therefore, suggest that TCR usage may be an important distinguishing factor between YeiH.232-240–specific CD8^+^ T cells in healthy individuals and patients with B27AAU or axSpA.

TCRs with the HLA-B*27 axSpA motif were uniformly found within the CD161^int^CD8^+^ T cell population. Clonally expanded CD8^+^ T cells in the eye in other forms of uveitis, however, did not express *KLRB1*, suggesting a pathogenic role specific to B27AAU. This suggests that the pathogenic mechanisms of B27AAU versus non-HLA-B*27–associated uveitis are distinct, which is supported by distinct genetics associated with B27AAU and HLA-B*27^–^ AAU (38). By contrast, CD161^int^CD8^+^ T cells have been suggested to have a role in other systemic autoimmune conditions, such as vasculitis (39), psoriatic arthritis (40), and multiple sclerosis (41). Future investigations will likely determine whether the CD161^int^CD8^+^ T cells in these autoimmune conditions contain cross-reactive TCRs that propagate inflammatory disease similar to the axSpA motif and whether prior antigen-mediated activation and differentiation occurs at a mucosal surface. Moreover, if the usage of alternative TRBVs for pathogenic TCRs limits the efficacy of TRBV9 depletion, targeted depletion of the CD161^int^CD8^+^ T cell population may provide an alternative strategy for therapeutic intervention.

Intestinal inflammation has long been associated with axSpA; however, the molecular or cellular links between the gut and the spine (or eye) has remained unclear. Prior studies in animal models have suggested that intestinal T cells exit the gut, migrate into the joint, and contribute to inflammatory arthritis (42). Furthermore, prior work in axSpA identified an inverse correlation between the abundance of intraepithelial lymphocytes in the colon and the concentration of circulating lymphocytes in the blood (43), consistent with a model of intestinal lymphocyte egress from the gut into the systemic circulation (and potentially into inflamed joints thereafter). Various reports have suggested roles for conventional αβ T cells (17, 27, 37), γδ T cells (42, 44), MAIT cells (21, 45, 46), and innate lymphocytes, such as ILC3s (47), in axSpA. Here, we report that CD8^+^ T cells with the axSpA motif bare signatures of mucosal differentiation. We therefore hypothesize that mucosal antigen-specific CD8^+^ T cells have a key pathophysiologic role that links intestinal inflammation with axSpA and B27AAU.

Given the potential central role for YeiH-specific CD8^+^ T cells in B27AAU and axSpA pathogenesis, we hypothesize that they may allow for the development of biomarkers of disease. For example, measurement of YeiH-specific CD8^+^ T cells distinguished participants with axSpA and/or B27AAU from individuals acting as healthy controls, suggesting that quantifying pathogenic CD8^+^ T cells could be used to facilitate a clinical diagnosis. This may also help identify early stages of disease before the full array of clinical manifestations have emerged. Given the high degree of variation in frequency of YeiH-specific CD8^+^ T cells across individuals, this approach will likely need to be refined to more directly measure pathogenic CD8^+^ T cells and/or to account for biologic confounders, similar to the use of CRP levels to measure systemic autoimmunity after adjusting for obesity (48).

We noted that the TRBV5-5^+^ UV019.1 TCR had only modest reactivity to our previously reported list of autoantigens, including GPER1, PRPF3, and RNASEH2B. While this could be the result of limited sensitivity to weak TCR-peptide-MHC interactions in our in vitro system, it is also possible that there exist additional autoantigens that may serve as ligands in vivo (17). We anticipate future studies will be able to solidify the role of specific autoantigens in B27AAU and axSpA.

Although YeiH.232-240–specific CD8^+^ T cells were numerically more frequent in the blood during quiescent B27AAU compared with a recent flare, this difference did not reach statistical significance due to the high degree of variation within each group. This difference could be explained by pathogenic T cells trafficking out of the blood into inflamed tissues during periods of flare; however, future studies with longitudinal sampling would be better suited to address this question.

Although the data presented here are based on a small collection of ocular samples from B27AAU and synovial samples from axSpA, the comparison with a larger non–HLA-B*27–associated uveitis cohort reinforces a shared pathophysiology of mucosal T cell infiltration. Furthermore, we were able to validate observations from the ocular and synovial scRNA-Seq data with flow cytometry of the peripheral blood in a larger cohort. Moreover, our finding that YeiH.232-240–specific CD8^+^ T cells are preferentially expanded in the peripheral blood of participants with axSpA and B27AAU corroborates the importance of this cell population in the pathophysiology of axSpA and B27AAU. Several questions remain, however, such as which intestinal compartment YeiH.232-240–specific CD8^+^ T cells occupy in the healthy state and whether this is disrupted during axSpA and B27AAU. In addition, the capacity to detect YeiH.232-240–specific CD8^+^ T cells in the peripheral blood will facilitate future prospective studies to determine whether the emergence of YeiH.232-240–specific CD8^+^ T cells precedes disease onset, similar to the emergence of autoantibodies in lupus and rheumatoid arthritis (49, 50).

## Methods

### Sex as a biological variable.

Both male and female participants were enrolled in this research study.

### Participants.

All participants with B27AAU met the Standardization of Uveitis Nomenclature Working Group classification criteria for B27AAU (51). In addition, 1 participant with B27AAU had r-axSpA, also known as ankylosing spondylitis, 1 had nr-axSpA, and 2 had isolated uveitis without systemic disease after a rheumatologic evaluation (52). Of the 5 B27AAU ocular samples, 4 were collected within 0–2 days of the initial encounter with ophthalmology (UV019a, UV027b, UV122a, and UV180a), while 1 (UV027a) was collected during relapsed inflammation after an initial 1-month course of oral corticosteroids followed by a self-reported 2–3 days of nonadherence. All B27AAU ocular samples were collected in the context of topical corticosteroids, with 50% on a TNF inhibitor (Table 3).

For ocular samples, participants with active anterior uveitis, defined by at least 1+ anterior chamber (AC) cell (>6 cells per high-powered field) (53), were enrolled for paired ocular and blood sampling. Participants with B27AAU were HLA-B*27^+^ by clinical laboratory testing. Participants with alternative etiologies for uveitis, including HLA-B*27^–^ anterior uveitis, anterior/intermediate uveitis, or pan-uveitis, were labeled as non-B27AAU participants. One participant in the non-B27AAU group was HLA-B*27^+^ but had a history of multifocal choroiditis. The remaining participants in the non-B27AAU group were HLA-B*27^–^ either by clinical laboratory testing or by research laboratory testing via PCR of isolated genomic DNA using previously validated HLA-B*27–specific primers (54). A recent B27AAU flare was defined as evidence of disease activity with 0.5+ AC cells or more on ophthalmologic exam within 35 days of symptom onset. Quiescent disease was defined as 0 AC cells on ophthalmologic exam, absence of B27AAU symptoms, and greater than 35 days since a previous B27AAU flare.

For blood samples, healthy participants were recruited by individual investigators or through the Volunteers for Health registry (Washington University in St. Louis) and were free of any autoimmune disease. For flow cytometry, participants in the B27AAU group had an established clinical diagnosis of B27AAU with a confirmatory positive HLA-B*27 test by research laboratory PCR. Participants with axSpA met the 2009 ASAS classification criteria for either r-axSpA or nr-axSpA (52). All participants with axSpA were HLA-B*27^+^ by research laboratory PCR. For samples classified with a B27AAU flare, participants had a clinic visit within approximately 1 month of sampling that documented active AC inflammation.

For synovial fluid samples, 2 participants with r-axSpA and active knee arthritis were recruited during routine clinical care as previously described (55). Both patients were HLA-B27^+^ and naive to conventional and biologic disease-modifying antirheumatic drugs. Synovial fluid samples were obtained during knee joint aspiration performed for therapeutic reasons.

### Biospecimen collection.

AC and blood sampling was performed as previously described (56, 57). Briefly, approximately 100–200 μL AC fluid was removed and centrifuged at 400*g* for 5 minutes, after which the aqueous fluid was removed. Blood samples were obtained by venipuncture, collected into EDTA tubes, and purified by Ficoll-Hypaque density gradient centrifugation. The AC cells and PBMCs were cryopreserved in FBS with 10% DMSO and stored at –140°C.

### scRNA-Seq.

scRNA-Seq was performed on paired eye and blood samples from the uveitis cohort (Table 3). This included 5 paired samples from 4 participants with B27AAU during a uveitis flare (17) and 14 paired samples from 14 nonparticipants with B27AAU, 4 of whom have been reported previously (56). We also collected 3 unpaired blood samples from individuals without uveitis as an internal reference during data processing. In total for this cohort, 41 samples underwent single-cell RNA sequencing (scRNA-Seq) (Supplemental Figure 5).

Frozen AC cells were thawed and washed once with FBS and once with PBS with 0.1% BSA. Due to low cell numbers (5,000–30,000 cells), minimal processing was performed to reduce cell loss. PBMCs were thawed and washed twice with 10% RPMI and then once with PBS with 0.1% BSA. Viability was >95% by Trypan Blue exclusion. Single-Cell 5′Gene Expression cDNA libraries were generated per the 10x Genomics Chromium Single-Cell 5′ Library and Gel Bead Kit v1 and the 10x Chromium Controller (10x Genomics) platform for microdroplet-based, single-cell barcoding, by the Genome Technology Access Center at the McDonnell Genome Institute (GTAC@MGI, Washington University in St. Louis). T cell and B cell enrichment libraries were generated per the Chromium Single-Cell V(D)J Enrichment Kits for Human T cell and Human B cell (10× Genomics) using the same input samples. All libraries were sequenced at GTAC@MGI on the NovaSeq Sequencing System (Illumina).

After thawing, synovial fluid cells were stained with Fixable Viability Dye eFluor520 (eBioscience) and the following antibodies: CD3-PerCP-Cy5.5 (OKT3), CD8a-PE (RPA-T4), CD45RA-PE/Dazzle (HI100), CD25-PE (BC96), and CD127-PE/Cy7 (A019D5) (Biolegend). Cells were then sorted on a Sony SH800Z prior to library preparation with the 10× Chromium Controller. Libraries were pooled and sequenced across multiple Illumina HiSeq 4000 lanes to obtain a read depth of approximately 30,000 reads per cell for gene-expression libraries and 8,500 reads per cell for V(D)J-enriched T cell libraries.

### Single-cell RNA expression analysis.

Sequencing reads were aligned to the human genome using Cell Ranger v3.1.0 for eye-blood samples, v6.1.1 for the CD161 sort and v8.1.0 for the YeiH-tetramer sort (10× Genomics). The publicly available Seurat R software package version 3.2.3 (58) was used for downstream analysis. Cells that had more than 11% of mitochondrial gene content were excluded from analysis. Doublets were identified using DoubletFinder v2.0.3 (59) and excluded from analysis. Samples were normalized using SCTransform (60). The SCT-transformed data were integrated with 3,000 anchor features and PrepSCTIntegration (https://www.rdocumentation.org/packages/Seurat/versions/3.1.3/topics/PrepSCTIntegration). A blood sample from a healthy participant was used as the reference data set to set integration anchors with FindIntegrationAnchors and to integrate the data with IntegrateData. Dimensionality reduction was done with runPCA using default settings and runUMAP with the top 30 calculated dimensions. Clustering was performed with a resolution of 2.5.

For the 211,696 cells in the uveitis data set, we identified 5 major immune cell types in the eye and blood (CD4^+^ T cell, CD8^+^ T cell, B cell, myeloid cell, or NK cell) based on canonical gene expression of the entire cluster (Supplemental Figure 5, B and C) as previously described (57). Clusters with genes from red blood cells (e.g., *HBA1*, *HBA2*, *HBB*), platelets (e.g., *PPBP*, *TUBB1*, *PF4*), or multiple cell types (e.g., *CD3D* and *CD79A*) were labeled as “other.” We focused our analysis on the 44,432 CD8^+^ T cells (Supplemental Figure 6), which were used for downstream analysis. Differential gene expression utilized Wilcoxon’s rank-sum test on normalized and scaled RNA count data. For differential gene expression across clusters, subclusters, or tissue-enriched clonotypes, FindAllMarkers function in the Seurat package was employed with a log-fold change threshold >0.25, minimum group percentage = 10%, and the pseudocount = 1. DEGs were defined as having an adjusted *P* value of less than 0.05 and a 1.5-fold increase in expression. Gene set enrichment analysis was performed using the escape R package (v1.3.1) (61). Gene sets were derived from the Hallmark library of the Molecular Signature Database (v7.0).

Similar to the uveitis data set, synovial CD8^+^ T cells were identified by expression of canonical markers and subsetted, followed by dimensionality reduction and clustering (Supplemental Figure 7).

### VDJ analysis.

T cell clonotypes were defined by Cell Ranger. Assignment of CD4^+^ or CD8^+^ T cell lineage to T cell clonotypes was based on whether the clonotype had a greater number of cells in the CD4^+^ or CD8^+^ T cell clusters, respectively. Evenly split clonotypes were labeled as “double positive” and not used for downstream calculations. To compare CD4^+^ and CD8^+^ T cell clonotype frequency across tissues, clonotypes were defined by having the same TRAV and TRBV gene segment usage with the same amino acid sequence for CDR3α and CDR3β. Clonotypes were assigned on the integrated Seurat object.

Expanded ocular clonotypes were defined as having more than 10 cells per clonotype within an ocular sample, as previously described (17). Clonotypes bearing the HLA-B*27 axSpA motif were defined as the (L/T) (Y/F)ST CDR3β motif paired with TRAV21; specifically *str-detect* was used to identify CDR3β containing the regular expression “^.{5,6}[L|T][Y|F]ST.{5}”.

### Peptide stimulation of T cell lines.

Transfection of SKW-3 T cells with the UV019.1 TCR and stimulation of transfected SKW-3 T cells with HLA-B*27:05–expressing K562 cells pulsed with bacterial and self-peptides was performed as previously described (17).

### Flow cytometry.

Frozen PBMCs were thawed and washed twice in RPMI + 10% FBS. 10 × 10^6^ cells were used for flow cytometric assessment of HLA-B*27–restricted CD8^+^ T cells specific to YeiH.232-240 (LRVMMLAPF) and NP.383-391 (SRYWAIRTR). YeiH.232-240 and NP.383-391 tetramers were generated as previously described (17). Cells were washed with PBS, stained with fixable viability dye (Thermo Fisher Scientific) at 4°C for 20 minutes, washed once with PBS, and centrifuged at 500*g* for 5 minutes. Cells were resuspended in Fc block (Thermo Fisher Scientific) for 10 minutes at room temperature and then stained with surface antibodies (all antibody reagents provided in Supplemental Table 4) for 30 minutes at room temperature. Cells were washed twice with 1% BSA in PBS and fixed with 2% PFA for flow cytometry.

Data were collected on a FACSCanto (BD Biosciences) or an Attune NxT Flow Cytometer (Thermo Fisher Scientific) and analyzed with FlowJo v10 (Tree Star).

### Peptide-based expansion of antigen-specific CD8^+^ T cells.

Expansion of NP.383-391 and YeiH.232-240–specific CD8^+^ T cells was performed as previously described (62). Briefly, PBMCs from HLA-B*27^+^ individuals were thawed as above in Gibco CTS OpTmizer Pro Serum Free Media (Thermo Fisher Scientific). 5 × 10^6^ PBMCs were used for each condition. Cells were labeled with CellTrace Violet (Thermo Fisher Scientific) per the manufacturer’s instructions for internal validation of proliferation. One-third of PBMCs for each sample were removed and pulsed with NP.383-391 or YeiH.232-240 at 10 μM for 90 minutes at 37°C. PBMCs were then recombined and cultured in Gibco CTS OpTmizer Pro Serum Free Media with Human IL-2 Recombinant Protein (Thermo Fisher Scientific) at 10 U/mL for 7–10 days with media exchange occurring every 2–3 days. For sorting of HLA-B*27(YeiH.232-240)^+^ CD8^+^ T cells after antigen-specific expansion, cultured PBMCs underwent CD8^+^ T cell enrichment with EasySep Human CD8^+^ T Cell Isolation Kit (StemCell), were washed with PBS, stained with LIVE/DEAD Fixable Near-IR Dead Cell Stain (Thermo Fisher Scientific), washed with PBS and 1% BSA, stained with TotalSeqC anti-human hashtag antibodies, washed twice with PBS and 1% BSA, and combined for staining with CD3, CD8, HLA-B*27(YeiH.232-240)-PE, and HLA-B*27(YeiH.232-240)-APC. Cells were sorted on CD3^+^, CD8^+^, PE/APC tetramer double-positive, and CellTrace Violet^–^ cells for single-cell TCR sequencing.

### TCR-pHLA-B*27 structural model generation.

AS8.4-YeiH-HLA-B*27 (PDB: 8CX4) and AS4.3-YeiH-HLA-B*27 (PDB: 7R2Q) were used to model the 026.1 and 135.1 TCR interaction with YeiH-HLA-B*27, respectively. These 2 models were chosen based on similar CDR3α length between AS8.4 and 026.1 and between AS4.3 and 135.1. The TCR models were generated by systemically mutating amino acids that are different from AS8.4 and AS4.3 to 026.1 and 135.1 in Pymol. The side chain conformation was selected based on the least steric hindrance formed with neighboring amino acids. The newly generated TCR-pHLA-B*27 models were used to prepare figures.

### Statistics.

Sample sizes were not predetermined using statistical methods. Investigators were not blinded to the experimental sample identities. For cell type frequency and flow cytometric analysis, ratio paired 2-tailed *t* test, unpaired 2-tailed *t* test, Holm-Šidák multiple 2-tailed *t* tests, Kruskal-Wallis test, 2-way ANOVA, and Dunn’s multiple comparisons test were performed using Prism software (GraphPad Software). For differential frequency of YeiH.232-240–specific CD8^+^ T cells during active or quiescent B27AAU, the *lmer* function from R package lme4 (v1.1-35.3) was used to calculate a linear mixed-effects model for continuous outcome. For differential gene expression, Wilcoxon’s rank-sum test from FindMarkers in Seurat was used. For single-cell gene set enrichment analysis, 2-tailed *t* test and 1-way ANOVA were performed with the escape package. *P* values of less than 0.0001 were considered significant.

### Study approval.

All human participants were enrolled in accordance with Declaration of Helsinki principles and using protocols approved by the institutional review board of Washington University in St. Louis (IRB 201704141, 201912043, 202011161, and 202212047) or South Central – Oxford C Research Ethics Committee (ethics reference 06/Q1606/139; Health Research Authority [Bristol], London, United Kingdom). Written informed consent was received from participants prior to inclusion in the study.

### Data availability.

Aligned sequencing data are available at figshare.com (https://doi.org/10.6084/m9.figshare.22078493.v1, https://doi.org/10.6084/m9.figshare.25796431.v1, and https://doi.org/10.6084/m9.figshare.25796431.v1). Values for all data points in graphs are reported in the Supporting Data Values file. Clinical information was stored in a REDCap database (https://www.project-redcap.org/). Deidentified data will be available made upon reasonable request.

## Author contributions

MAP, XY, KCG, and WMY designed the study, analyzed the data, and wrote the manuscript. MAP, LMH, GLP, NL, PHADF, AF, SYL, DS, LFL, LES, CL, and PB identified and recruited participants and controls and collected biospecimens. MAP, LMH, FP, NL, PHADF, JL, and LES processed biospecimens. MAP, FP, LIG, EDOR, PAR, EE, and GMG performed analysis of single-cell RNA sequencing data. MAP, NL, and RA performed flow cytometry experiments. XY performed TCR stimulation assays for the UV019.1 TCR, synthesized tetramers for flow cytometry, and modeling of peptide-MHC-TCR interactions. MAP and XY are listed as co–first authors, with MAP listed first due to performing or supervising the larger half of experiments and analysis.

## Supplementary Material

Supplemental data

Supporting data values

## Figures and Tables

**Table 1:**
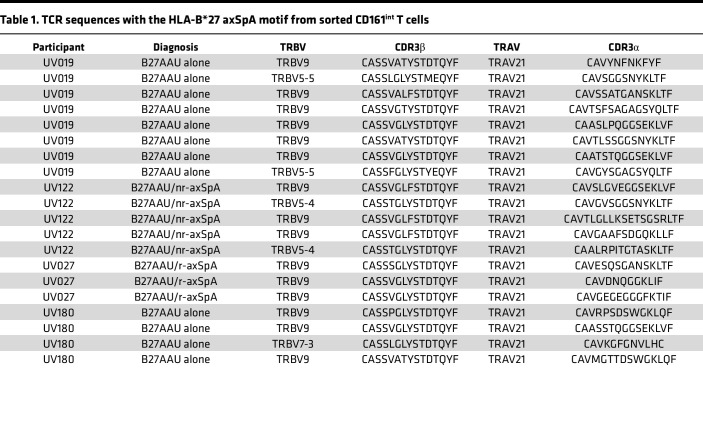
TCR sequences with the HLA-B*27 axSpA motif from sorted CD161^int^ T cells

**Table 2:**
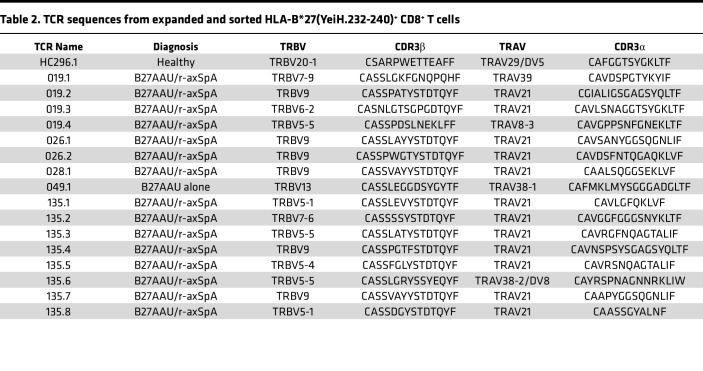
TCR sequences from expanded and sorted HLA-B*27(YeiH.232-240)^+^ CD8^+^ T cells

**Table 3:**
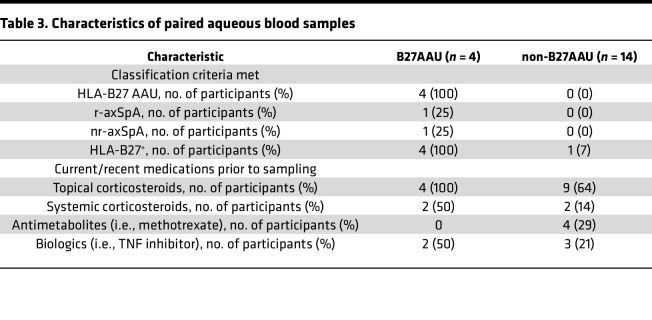
Characteristics of paired aqueous blood samples

## Version 1 07/18/2024

In-Press Preview

## Version 2 08/22/2024

Electronic publication

## References

[1] Robinson PC (2021). Axial spondyloarthritis: concept, construct, classification and implications for therapy. Nat Rev Rheumatol.

[2] Schlosstein L (1973). High association of an HL-A antigen, W27, with ankylosing spondylitis. N Engl J Med.

[3] Evans DM (2011). Interaction between ERAP1 and HLA-B27 in ankylosing spondylitis implicates peptide handling in the mechanism for HLA-B27 in disease susceptibility. Nat Genet.

[4] Baraliakos X, Braun J (2015). Non-radiographic axial spondyloarthritis and ankylosing spondylitis: what are the similarities and differences?. RMD Open.

[5] Brewerton DA (1973). Ankylosing spondylitis and HL-A 27. Lancet.

[6] Feltkamp TE, Ringrose JH (1998). Acute anterior uveitis and spondyloarthropathies. Curr Opin Rheumatol.

[7] Martin TM, Rosenbaum JT (2011). An update on the genetics of HLA B27-associated acute anterior uveitis. Ocul Immunol Inflamm.

[8] Standardization of Uveitis Nomenclature (sun) Working Group (2021). Classification criteria for spondyloarthritis/HLA-B27-associated anterior uveitis. Am J Ophthalmol.

[9] Chang JH (2005). Acute anterior uveitis and HLA-B27. Surv Ophthalmol.

[10] Laurence M (2018). Spondyloarthritis, acute anterior uveitis, and fungi: updating the Catterall-King hypothesis. Front Med (Lausanne).

[11] Bowness P (2015). HLA-B27. Annu Rev Immunol.

[12] DeLay ML (2009). HLA-B27 misfolding and the unfolded protein response augment interleukin-23 production and are associated with Th17 activation in transgenic rats. Arthritis Rheum.

[13] Mielants H (1988). Ileocolonoscopic findings in seronegative spondylarthropathies. Br J Rheumatol.

[14] Dworkin MS (2001). Reactive arthritis and Reiter’s syndrome following an outbreak of gastroenteritis caused by Salmonella enteritidis. Clin Infect Dis.

[15] Hannu T (2005). Reactive arthritis attributable to Shigella infection: a clinical and epidemiological nationwide study. Ann Rheum Dis.

[16] Rosenbaum JT, Asquith M (2018). The microbiome and HLA-B27-associated acute anterior uveitis. Nat Rev Rheumatol.

[17] Yang X (2022). Autoimmunity-associated T cell receptors recognize HLA-B*27-bound peptides. Nature.

[18] Britanova OV (2023). Targeted depletion of TRBV9^+^ T cells as immunotherapy in a patient with ankylosing spondylitis. Nat Med.

[19] Nasonov EL (2024). Effectiveness and safety of BCD180, anti-TRBV9+ T-lymphocytes monoclonal antibody in patients with active radiographic axial spondyloarthritis: 36-week results of double-blind randomized placebo-controlled phase II clinical study ELEFTA. Rheumatology Science and Practice.

[20] Godfrey DI (2019). The biology and functional importance of MAIT cells. Nat Immunol.

[21] Gracey E (2016). IL-7 primes IL-17 in mucosal-associated invariant T (MAIT) cells, which contribute to the Th17-axis in ankylosing spondylitis. Ann Rheum Dis.

[22] Huang JC (2021). Preliminary report on interleukin-22, GM-CSF, and IL-17F in the pathogenesis of acute anterior uveitis. Ocul Immunol Inflamm.

[23] Gherardin NA (2018). The diverse family of MR1-restricted T Cells. J Immunol.

[24] Fergusson JR (2016). CD161(int)CD8+ T cells: a novel population of highly functional, memory CD8+ T cells enriched within the gut. Mucosal Immunol.

[25] Fergusson JR (2014). CD161 defines a transcriptional and functional phenotype across distinct human T cell lineages. Cell Rep.

[26] FitzPatrick MEB (2021). Human intestinal tissue-resident memory T cells comprise transcriptionally and functionally distinct subsets. Cell Rep.

[27] Faham M (2017). Discovery of T cell receptor β motifs specific to HLA-B27-positive ankylosing spondylitis by deep repertoire sequence analysis. Arthritis Rheumatol.

[28] Hanson AL (2020). Altered repertoire diversity and disease-associated clonal expansions revealed by T cell receptor immunosequencing in ankylosing spondylitis patients. Arthritis Rheumatol.

[29] Zheng M (2019). TCR repertoire and CDR3 motif analyses depict the role of αβ T cells in Ankylosing spondylitis. EBioMedicine.

[30] Xiao Z (2007). Detuning CD8 T cells: down-regulation of CD8 expression, tetramer binding, and response during CTL activation. J Exp Med.

[31] Wang C (2009). The roles of CCR6 in migration of Th17 cells and regulation of effector T-cell balance in the gut. Mucosal Immunol.

[32] Kieskamp SC (2021). Central sensitization, illness perception and obesity should be considered when interpreting disease activity in axial spondyloarthritis. Rheumatology (Oxford).

[33] Wakefield D (2020). Recent developments in HLA B27 anterior uveitis. Front Immunol.

[34] Feagan BG (2013). Vedolizumab as induction and maintenance therapy for ulcerative colitis. N Engl J Med.

[35] Sandborn WJ (2013). Vedolizumab as induction and maintenance therapy for Crohn’s disease. N Engl J Med.

[36] Komech EA (2022). TCR repertoire profiling revealed antigen-driven CD8+ T cell clonal groups shared in synovial fluid of patients with spondyloarthritis. Front Immunol.

[37] Dulphy N (1999). Common intra-articular T cell expansions in patients with reactive arthritis: identical beta-chain junctional sequences and cytotoxicity toward HLA-B27. J Immunol.

[38] Gelfman S (2023). A large meta-analysis identifies genes associated with anterior uveitis. Nat Commun.

[39] Klapa S (2021). Expansion of CD161 expressing CD8+ single-positive and CD4+CD8+ double-positive PR3-specific T-cells in granulomatosis with polyangiitis. Clin Exp Rheumatol.

[40] Povoleri GAM (2023). Psoriatic and rheumatoid arthritis joints differ in the composition of CD8+ tissue-resident memory T cell subsets. Cell Rep.

[41] Nicol B (2018). An intermediate level of CD161 expression defines a novel activated, inflammatory, and pathogenic subset of CD8+ T cells involved in multiple sclerosis. J Autoimmun.

[42] Lefferts AR (2022). Cytokine competent gut-joint migratory T Cells contribute to inflammation in the joint. Front Immunol.

[43] Regner EH (2018). Functional intraepithelial lymphocyte changes in inflammatory bowel disease and spondyloarthritis have disease specific correlations with intestinal microbiota. Arthritis Res Ther.

[44] Kenna TJ (2012). Enrichment of circulating interleukin-17-secreting interleukin-23 receptor-positive γ/δ T cells in patients with active ankylosing spondylitis. Arthritis Rheum.

[45] Hayashi E (2016). Involvement of mucosal-associated invariant T cells in ankylosing spondylitis. J Rheumatol.

[46] Toussirot E (2018). Increased IL-22- and IL-17A-producing mucosal-associated invariant T cells in the peripheral blood of patients with ankylosing spondylitis. Front Immunol.

[47] Ciccia F (2015). Type 3 innate lymphoid cells producing IL-17 and IL-22 are expanded in the gut, in the peripheral blood, synovial fluid and bone marrow of patients with ankylosing spondylitis. Ann Rheum Dis.

[48] Visser M (1999). Elevated C-reactive protein levels in overweight and obese adults. JAMA.

[49] Arbuckle MR (2003). Development of autoantibodies before the clinical onset of systemic lupus erythematosus. N Engl J Med.

[50] Smolen JS (2018). Rheumatoid arthritis. Nat Rev Dis Primers.

[51] Standardization of uveitis nomenclature working G (2021). classification criteria for spondyloarthritis/HLA-B27-associated anterior uveitis. Am J Ophthalmol.

[52] Rudwaleit M (2009). The development of Assessment of SpondyloArthritis International Society classification criteria for axial spondyloarthritis, part II: validation and final selection. Ann Rheum Dis.

[53] Jabs DA (2005). Standardization of uveitis nomenclature for reporting clinical data. Results of the First International Workshop. Am J Ophthalmol.

[54] Sayer DC (1999). HLA-B*27 typing by sequence specific amplification without DNA extraction. Mol Pathol.

[55] Simone D (2021). Single cell analysis of spondyloarthritis regulatory T cells identifies distinct synovial gene expression patterns and clonal fates. Commun Biol.

[56] Hassman LM (2021). Clinicomolecular identification of conserved and individualized features of granulomatous uveitis. Ophthalmol Sci.

[57] Paley MA (2022). The CSF in neurosarcoidosis contains consistent clonal expansion of CD8 T cells, but not CD4 T cells. J Neuroimmunol.

[58] Stuart T (2019). Comprehensive integration of single-cell data. Cell.

[59] (2019). DoubletFinder: doublet detection in single-cell RNA sequencing data using artificial nearest neighbors. Cell Syst.

[60] Hafemeister C, Satija R (2019). Normalization and variance stabilization of single-cell RNA-seq data using regularized negative binomial regression. Genome Biol.

[61] Borcherding N (2021). Mapping the immune environment in clear cell renal carcinoma by single-cell genomics. Commun Biol.

[62] Grant EJ, Gras S (2022). Protocol for generation of human peptide-specific primary CD8+ T cell lines. STAR Protoc.

