# Supplementary material for: Mucosal signatures of pathogenic T cells in HLA-B*27+ anterior uveitis and axial spondyloarthritis
Source: JCI Insight. 2024 Jul 18;9(16):e174776. doi: 10.1172/jci.insight.174776 (PMC11343591; doi:10.1172/jci.insight.174776)
Supplement: Supplemental data [file jciinsight-9-174776-s253.pdf]

SUPPLEMENTAL DATA

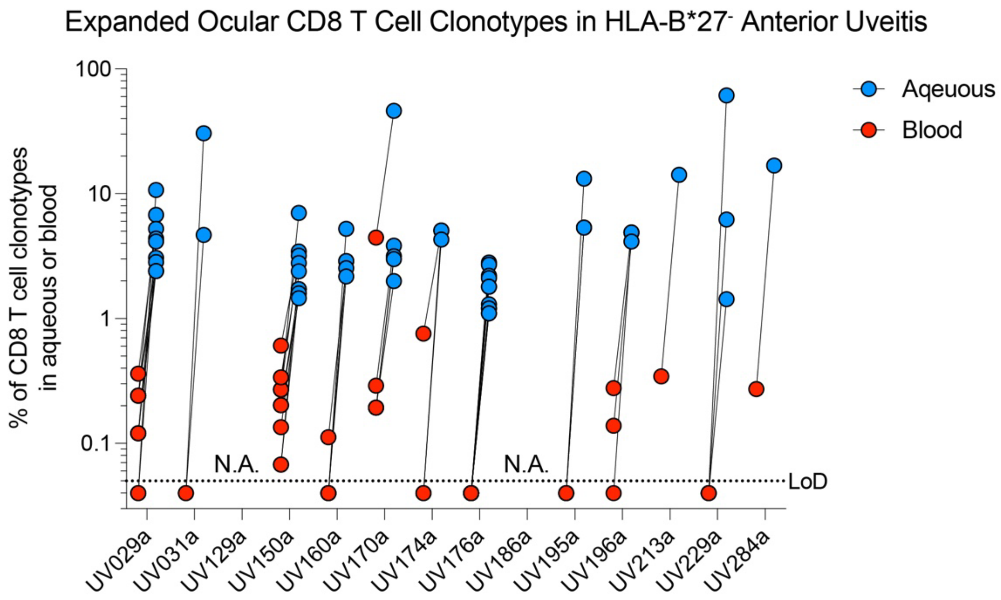

Supplemental Figure 1. The majority of HLA-B*27^{-} anterior uveitis ocular samples contain clonally expanded CD8 T cells. Percent of CD8 T cells in the eye or blood for expanded ocular CD8 T cell clonotypes. Expanded ocular CD8 T cell clonotypes were defined as having 10 or more barcodes in the eye and a 5-fold increase in frequency in the eye over the blood. Of the fourteen non-B27AAU ocular samples, twelve had expanded CD8 T cell clonotypes. The level of detection (LoD) of blood samples is indicated by a dashed line and is the median proportion of a singleton clonotype from all blood samples. N.A., not applicable.

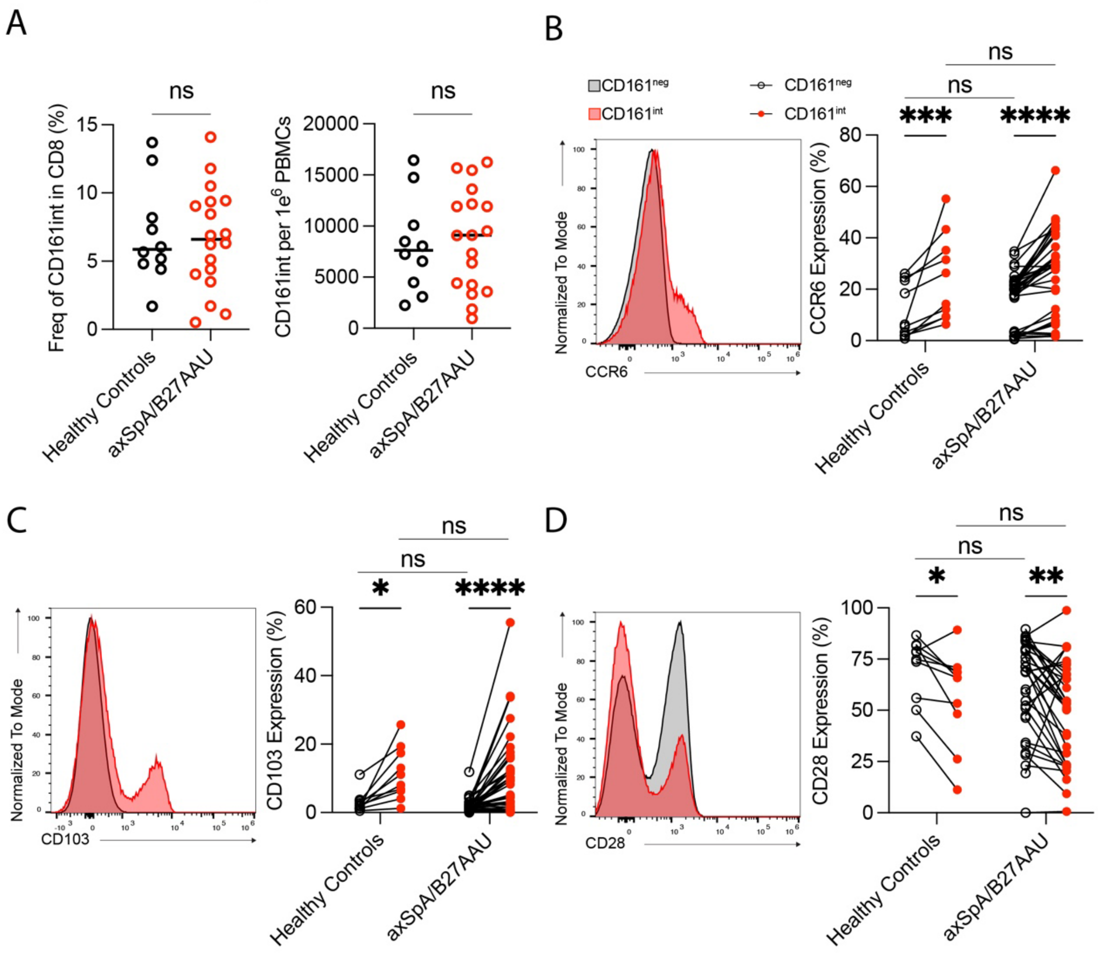

Supplemental Figure 2. Differentiation into CD161^{int} CD8 T cells may account for some, but not all HLA-B*27 axSpA motif DEGs. (A) Quantification of CD161^{int} CD8 T cells in the blood of HLA-B*27^{+} healthy participants and participants with axSpA and/or B27AAU. Cells are gated on CD4^{-}CD19^{-}Vα7.2^{-}Vα24.Jα18^{-}CD3^{+}CD8^{+} T cells. (B-D) Expression of CCR6 (B), CD103 (C), and CD28 (D) on CD161^{int} and CD161^{neg} CD8 T cells in the blood of HLA-B*27^{+} healthy participants and axSpA/ B27AAU participants. *, $p < 0.05$; **, $p < 0.01$; ***, $p < 0.001$; ****, $p < 0.0001$; ns, not significant; *t* test (Panel A), 2-way ANOVA (Panels B, C, & D)

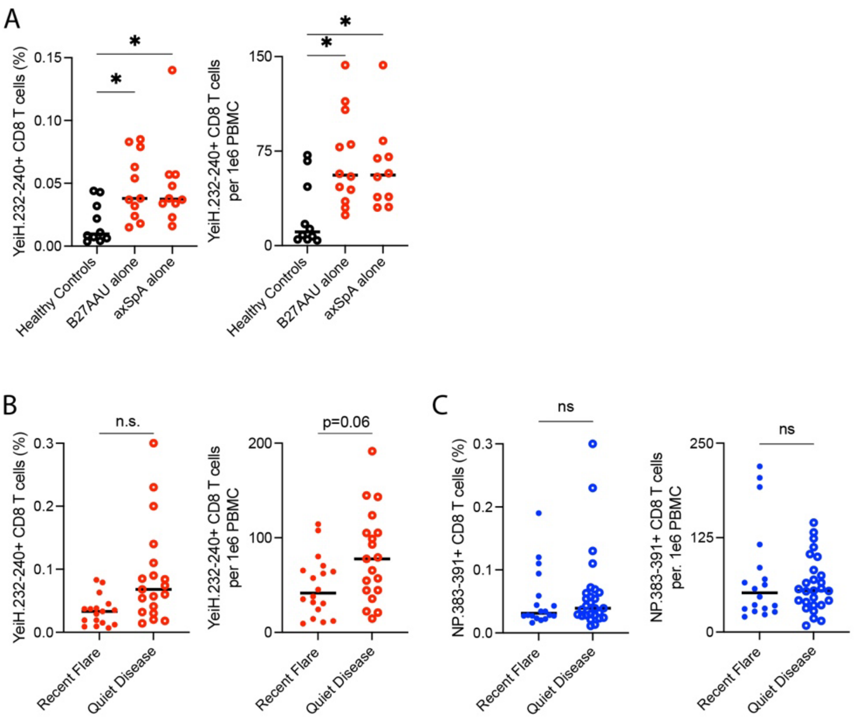

Supplemental Figure 3. YeiH.232-240-specific CD8 T cells are expanded in participants with B27AAU or axSpA alone and are numerically increased in participants with quiescent uveitis. (A) Quantification of HLA-B*27(YeiH.232-240)$^{+}$ CD8 T cells in HLA-B*27^{+} healthy controls from Figure 4B compared to participants with HLA*B27^{+} axSpA alone or B27AAU alone. (B-C) Quantification of HLA-B*27(YeiH.232-240)$^{+}$ (B) or HLA-B*27(NP.383-391)$^{+}$ (C) CD8 T cells within 35 days of a B27AAU flare or during a period of disease quiescence. *, $p < 0.05$, Kruskal-Wallis with Dunn's multiple comparisons test (Panel A), linear mixed effects model for continuous outcome (Panels B & C).

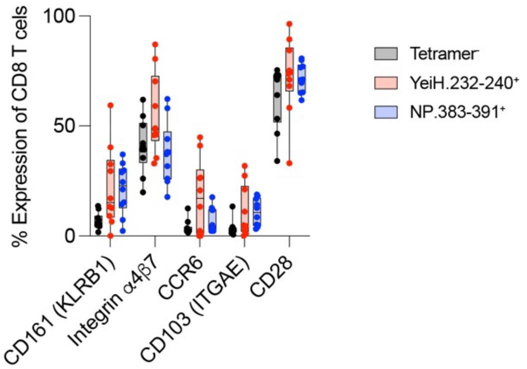

Supplemental Figure 4. Analysis of YeiH.232-240-specific CD8 T cells in HLA-B*27$^+$ healthy controls. Expression of CD161, integrin α4β7, CCR6, CD103, and CD28 on HLA-B*27(YeiH.232-240)$^+$ (red), HLA-B*27(NP.383-391)$^+$ (blue), and tetramer negative (black) CD8 T cells.

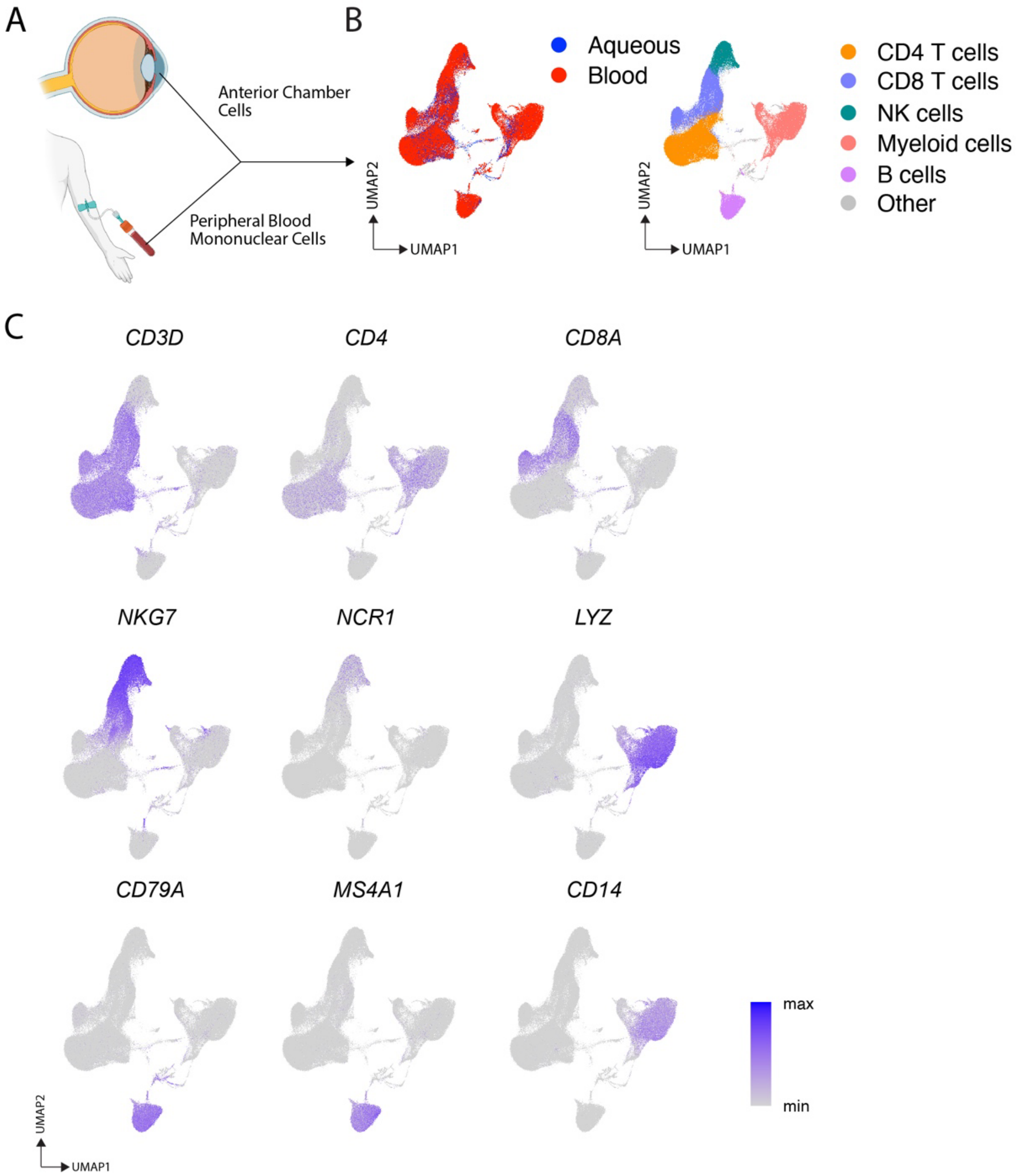

Supplemental Figure 5. Workflow for single cell RNA sequencing of paired ocular-blood samples. (A) Cells were collected from the anterior chamber and blood for scRNAseq. (B) UMAP plot of all samples colored by tissue source (left) and major cell lineage (right). (C) Expression of indicated genes to identify T cells (CD3D), B cells (CD79A, MS4A1), NK cells (NCR1, NKG7), and myeloid cells (LYZ, CD14). CD4 and CD8 T cell clusters were distinguished by CD4 or CD8A expression, respectively.

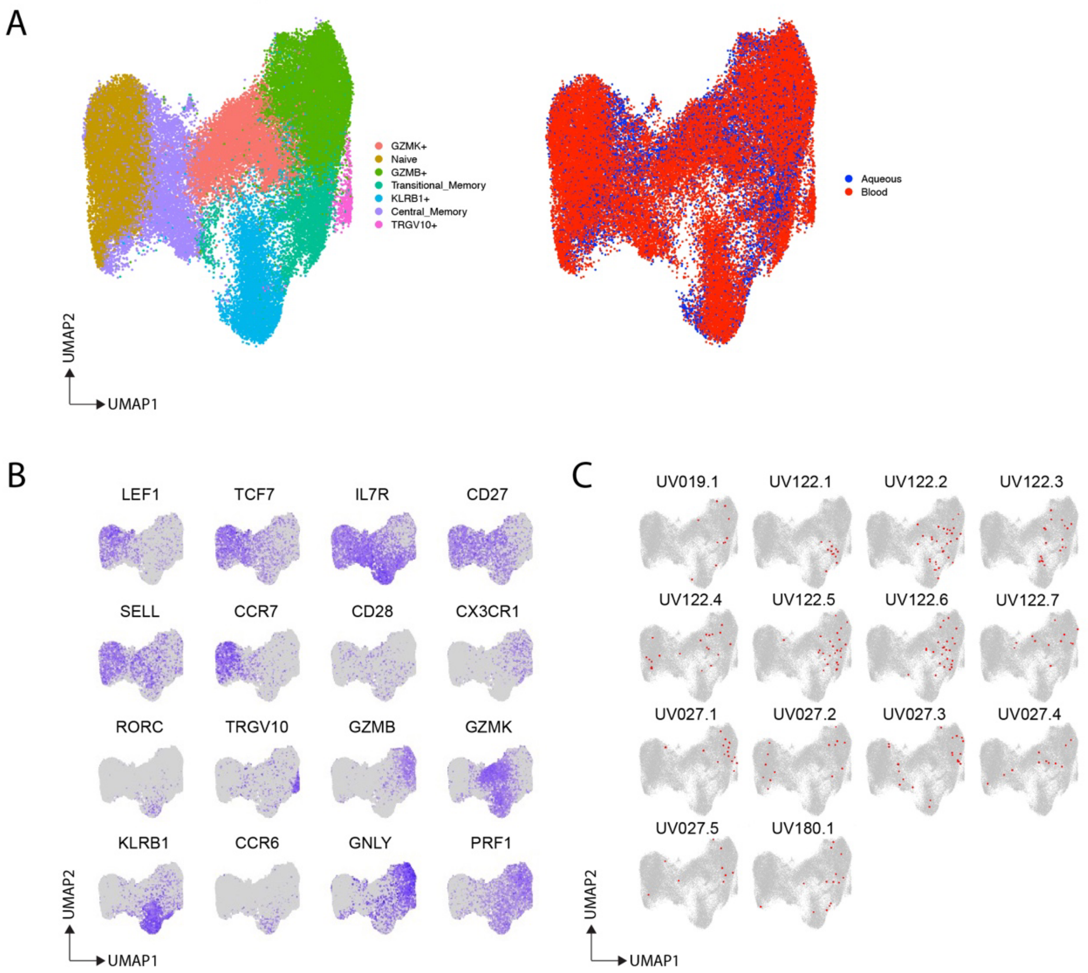

Supplemental Figure 6. Analysis of CD8 T cells from paired eye-blood samples. (A) UMAP plot of CD8 T cells from aqueous and blood, colored and annotated by cluster (left) or tissue (right). (B) Canonical gene expression for lineage annotation in (A). (C) Highlighting of expanded CD8 T cell clonotypes from B27AAU from Figure 1A.

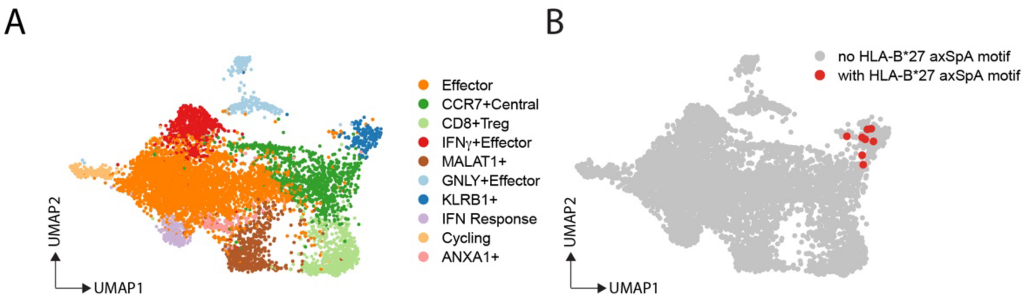

Supplemental Figure 7. Analysis of synovial CD8 T cells from HLA-B27^{+} axSpA. (A) UMAP plot of CD8 T cells from synovial fluid of two axSpA participants, colored and annotated by cluster. (B) Highlighting of CD8 T cells bearing the axSpA motif [defined as pairing of TRAV21 with a (L/T)(Y/F)ST CDR3b motif].

Supplemental Table 1. TCR sequences of expanded CD8 T cells in aqueous fluid of B27AAU.

Subject_Timepoint	TRBV	CDR3b	TRAV	CDR3a
UV019_a	TRBV5-5	CASSLGLYSTMEQYF	TRAV21	CAVSGGSNYKLTF
UV027_b	TRBV15	CATIQDSYNEQFF	TRAV9-2	CASDGGTDKLIF
UV027_b	TRBV19	CAAQGGANTEAFF	TRAV13-1	CAASGYSTLTF
UV027_b	TRBV19	CASSMNTGELFF	TRAV13-1	CAARPGGYNKLIF
UV027_b	TRBV2	CASRPGEPYEQYF	TRAV8-4	CAVISLSGGYNKLIF
UV027_b	TRBV24-1	CATSDLGGYGYTF	TRAV8-4	CAVTYSSASKIIF
UV122_a	TRBV29-1	CSVALYNEQFF	TRAV10	CVVSRTTSGTYKYIF
UV122_a	TRBV29-1	CSVVQETQYF	TRAV8-4	CAVSGGWGARLMF
UV122_a	TRBV5-1	CASRRDLGTSSVEQFF	TRAV5	CAERAARQLTF
UV122_a	TRBV6-1	CASSEDQGSSYEQYF	TRAV16	CAQPGTDKLIF
UV122_a	TRBV6-1	CASSPLGASSYEQYF	TRAV24	CALQGARLMF
UV122_a	TRBV7-2	CASSSDGGFSNEQFF	TRAV6	CALVMDSNYQLIW
UV122_a	TRBV7-2	CASSSDGGFSNEQFF	TRAV8-6	CALRSSGAGSYQLTF
UV122_a	TRBV7-9	CASSPRTAMNTEAFF	TRAV5	CAESPAMDTGRRALTF
UV180_a	TRBV9	CASSPGLYSTDTQYF	TRAV21	CAVRPSDSWGKLQF
UV180_a	TRBV9	CASSVATYSTDTQYF	TRAV21	CAVMGTTDSWGKLQF

Supplemental Table 2. Quality Metrics of scRNAseq from ocular-blood paired samples and synovial samples.

	Eye samples	Blood Samples	Synovial Fluid Samples	CD161int T cells	CD161neg T cells
**Median Number of Barcodes Per Sample**	1657	7204	3724	7003	5771
**Median Number of Genes per Cell**	1684	1559	1124	1531	1989
**Median Number of UMI per Cell**	4852	4546	2663	3835	5356

Supplemental Table 3. Medication usage of B27AAU participants during active or quiescent disease

Medication - n (%)	Active B27AAU (n=12)	Quiescent B27AAU (n=21)
None	3 (25)	5 (24)
Topical Steroids	5 (42)	3 (14)
NSAIDs	1 (8)	6 (29)
MTX	1 (8)	0 (0)
TNFi	1 (8)	8 (38)
JAKi	1 (8)	2 (10)

Supplemental Table 4. Antibodies used for flow cytometry.

Target	Conjugate	Clone	Vendor	Catalog Number
Integrin a4b7	AF488	Hu117	R&D Systems	FAB10078G
CD8	FITC	HIT8α	BD Biosciences	555634
MR1	PE	n/a	NIH Tetramer Core	n/a
CD19	PE-Dazzle594	HIB19	Biolegend	302252
TCRgd	PE-Dazzle594	B1	Biolegend	331225
TCR Va7.2	PE-Dazzle594	3C10	Biolegend	351730
Va24-Ja18	PE-Dazzle594	6B11	Biolegend	342920
CD161	PerCP-Cy5.5	HP-3G10	ThermoFisher	45-1619-42
CD19	APC	HIB19	Biolegend	302212
Vα7.2	AF700	3C10	Biolegend	351728
TCR$\alpha\beta$	BV421	IP26	Biolegend	306722
CD4	eF506	RPA-T4	ThermoFisher	69-0049-42
CCR6	BV510	G034E3	Biolegend	353424
CD28	eF540	CD28.2	ThermoFisher	48-0289-42
CD3	SB600	OKT3	ThermoFisher	63-0037-42
CCR6	BV650	G034E3	Biolegend	353426
CD103	BV650	Ber-ACT8	BD Biosciences	743653
CD4	BV711	RPA-T4	Biolegend	300558
CD8a	BV785	RPA-T8	Biolegend	301046
